# Supplementary figures and images for: Population-level genome-wide STR discovery and validation for population structure and genetic diversity assessment of Plasmodium species
Source: PLoS Genet. 2022 Jan 10;18(1):e1009604. doi: 10.1371/journal.pgen.1009604 (PMC8782505; doi:10.1371/journal.pgen.1009604)

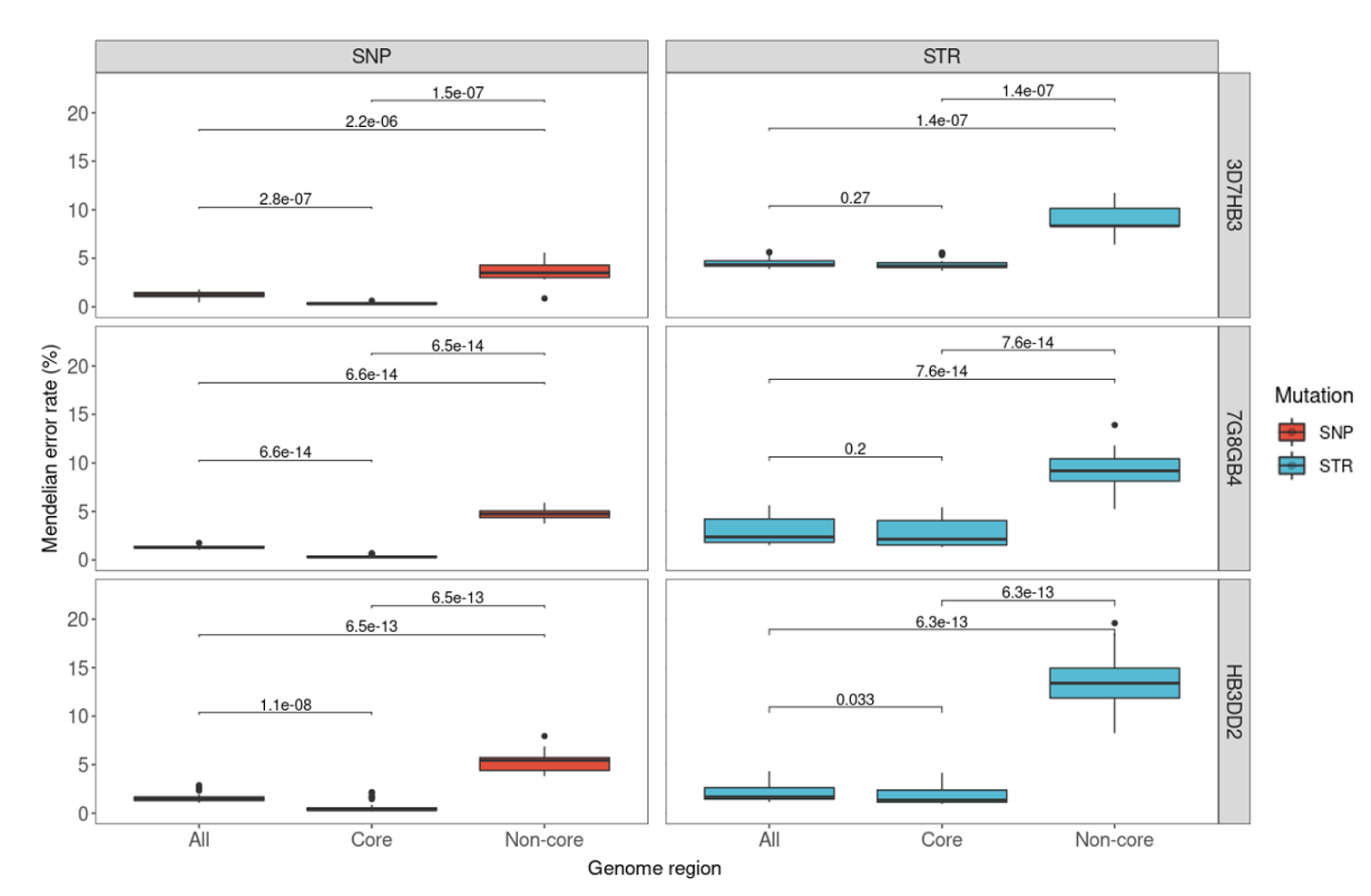

Supplement: S1 Fig — Test results (p-values) are from a two-sample Wilcoxon rank-sum test. (TIF) [file pgen.1009604.s001.tif]

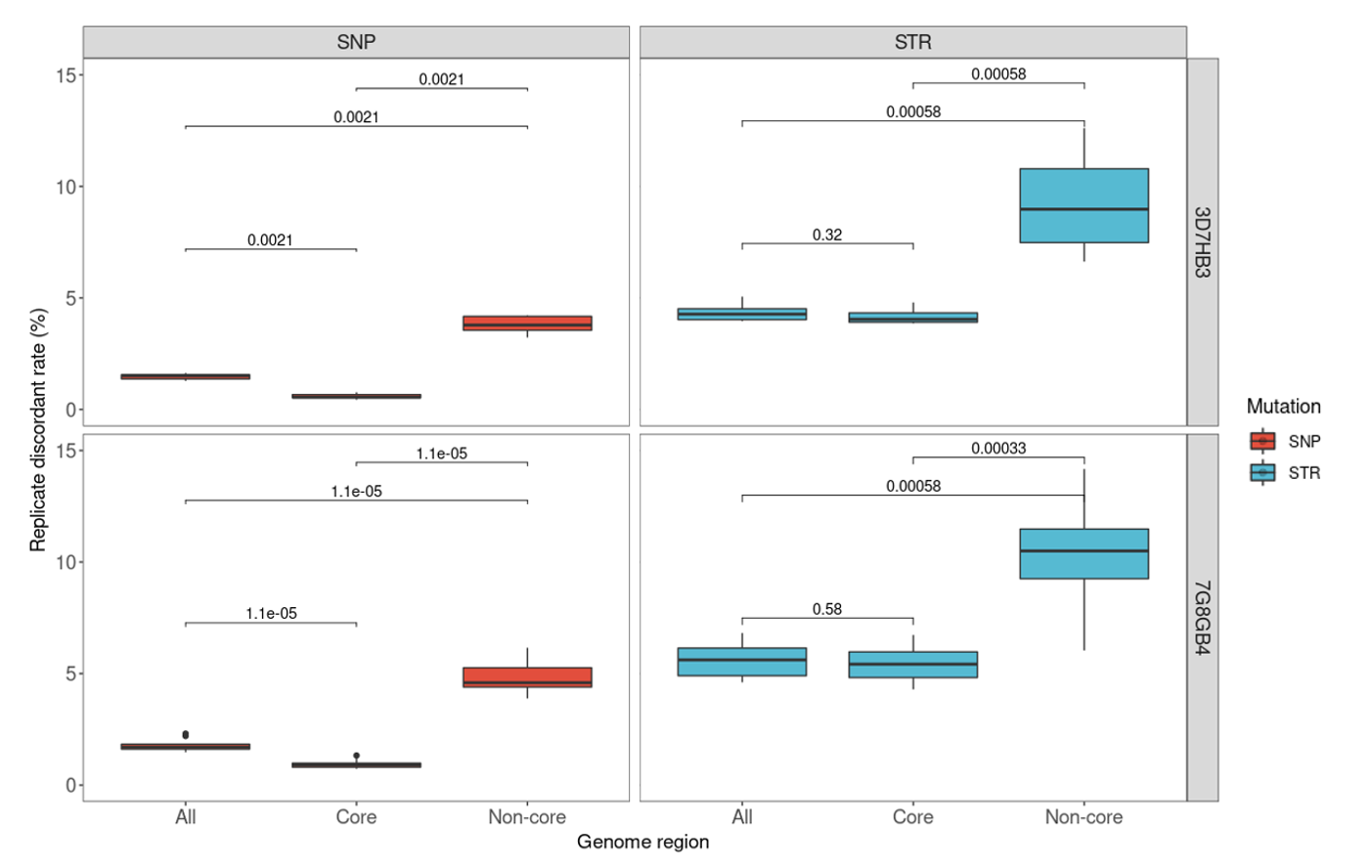

Supplement: S2 Fig — Test results (p-values) are from a two-sample Wilcoxon rank-sum test. (TIF) [file pgen.1009604.s002.tif]

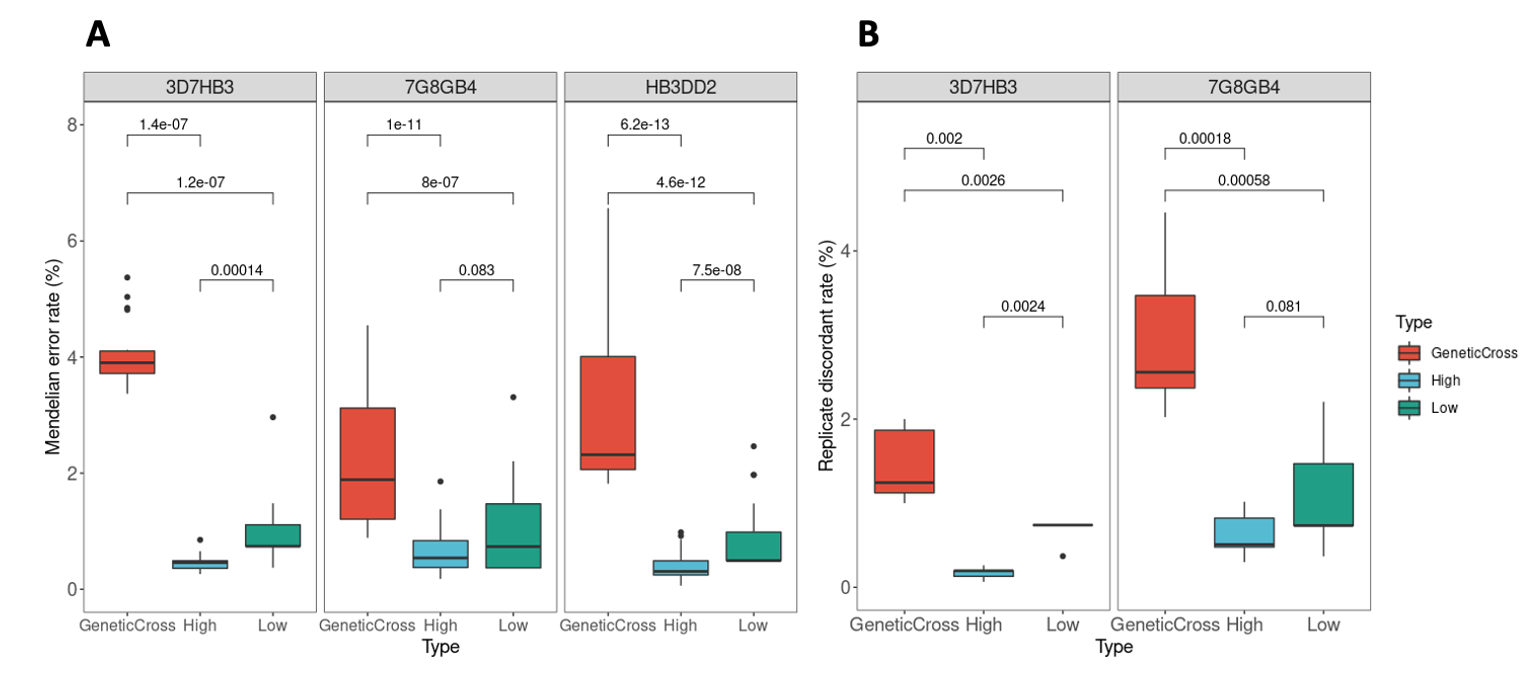

Supplement: S3 Fig — (A) The Mendelian error rate of STR genotypes. (B) Discordance rate of STR genotypes between biological replicates. Test results (p-values) are from a two-sample Wilcoxon rank-sum test. (TIF) [file pgen.1009604.s003.tif]

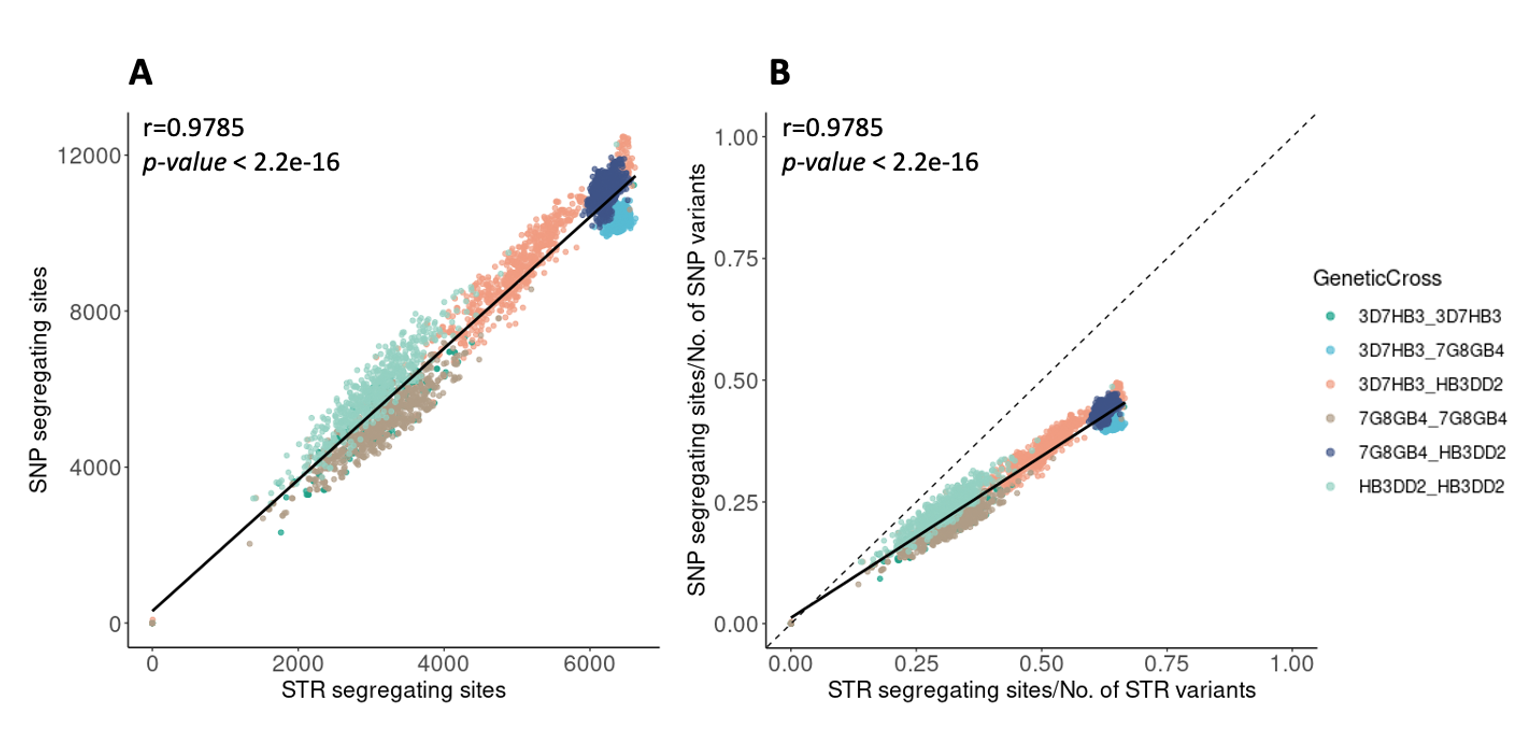

Supplement: S4 Fig — (A) The number of segregating sites between pairwise samples for both STRs and SNPs. (B) The relative number of segregating sites (segregating sites/No. of variants) between pairwise samples for both STRs and SNPs. The dashed line represents y = x. Pearson correlation coefficient r and test results (p-values) are indicated for each plot. (TIF) [file pgen.1009604.s004.tif]

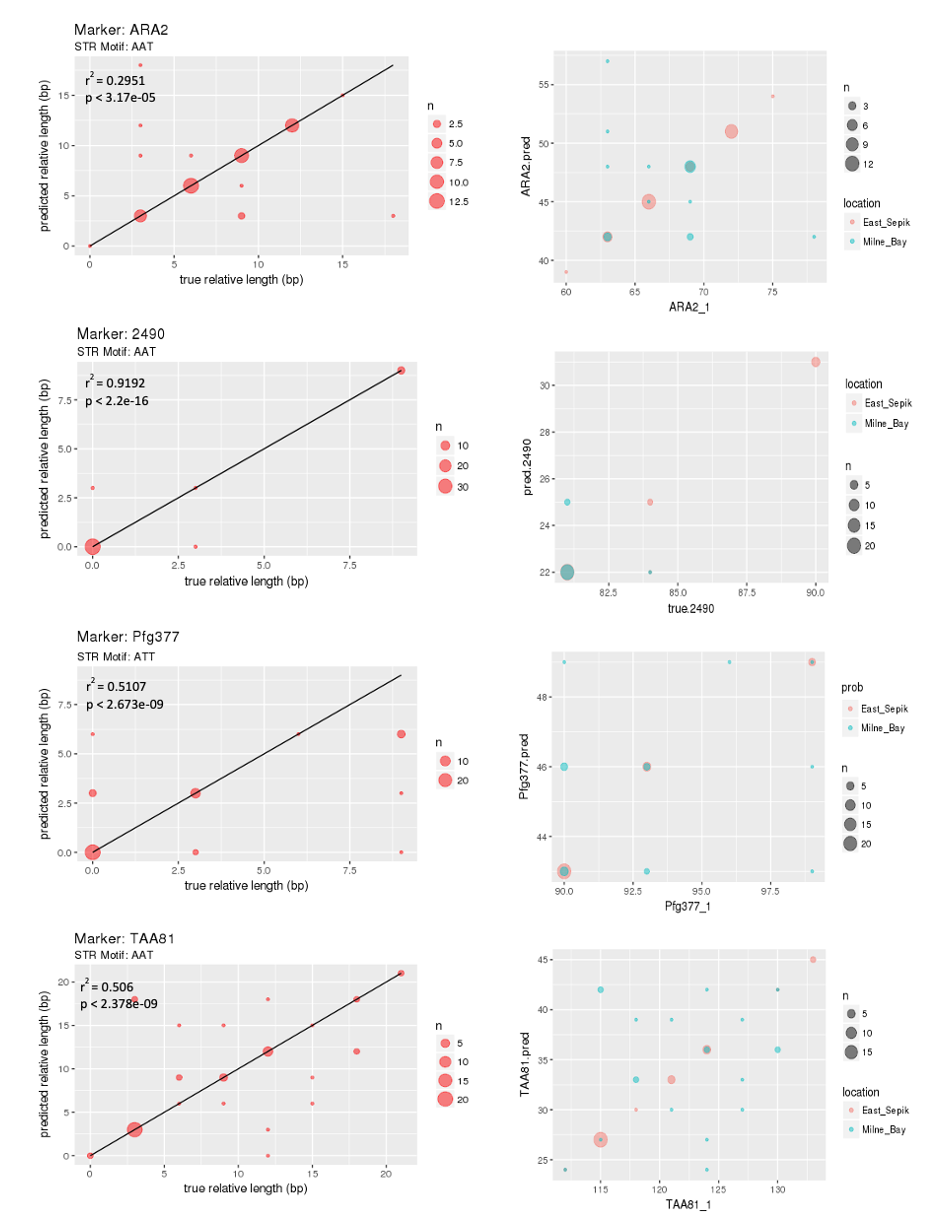

Supplement: S5 Fig — Bubble plots representing the GE allele calls are plotted against HipSTR’s calls, where the left plot has been shifted such that the bottom leftmost point lies on the origin. In the right plots points are coloured according to the region where samples originated from. The line represents y = x. It is important to note that in most of these plots, the Milne Bay samples are typically off the line y = x, indicating potential underlying issues with the GE calls for Milne Bay samples. (TIF) [file pgen.1009604.s005.tif]

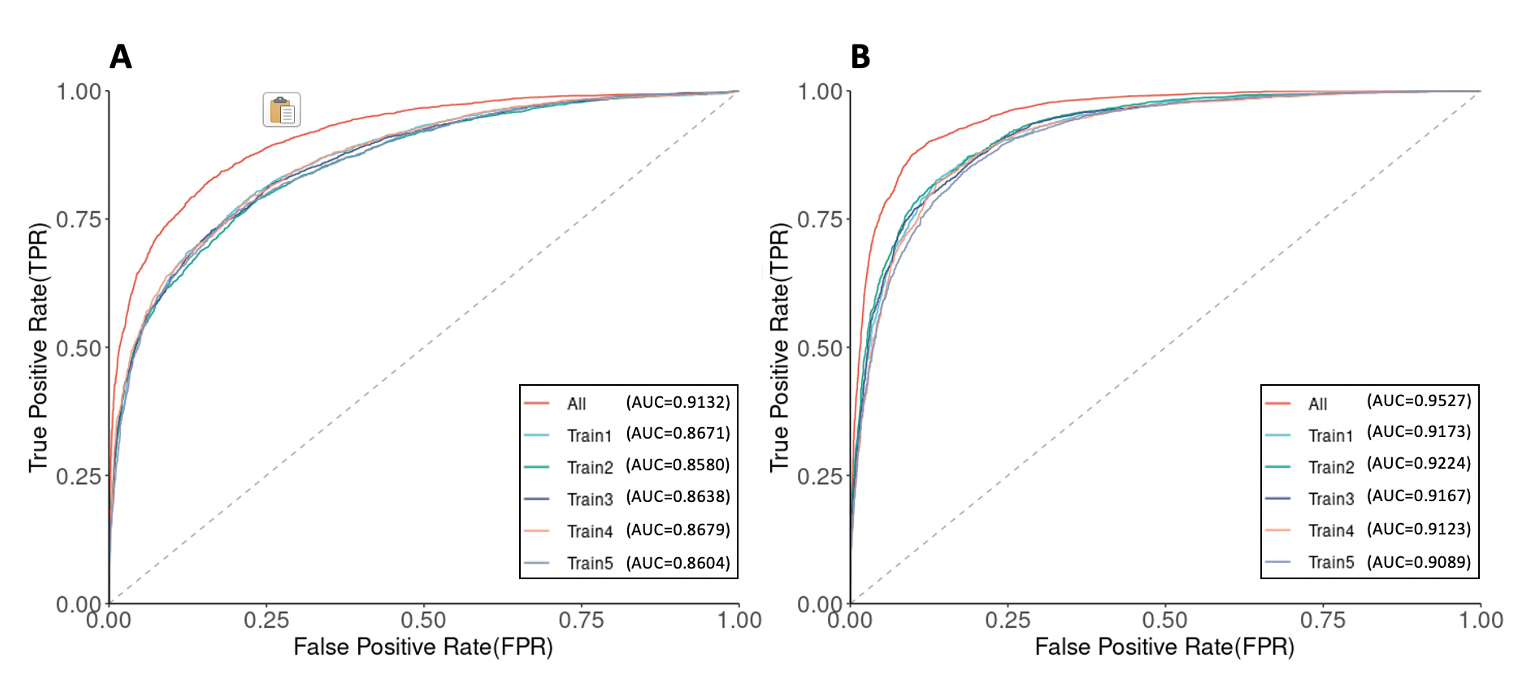

Supplement: S6 Fig — (A) The mononucleotide STR model. (B) The polynucleotide STR model. (TIF) [file pgen.1009604.s006.tif]

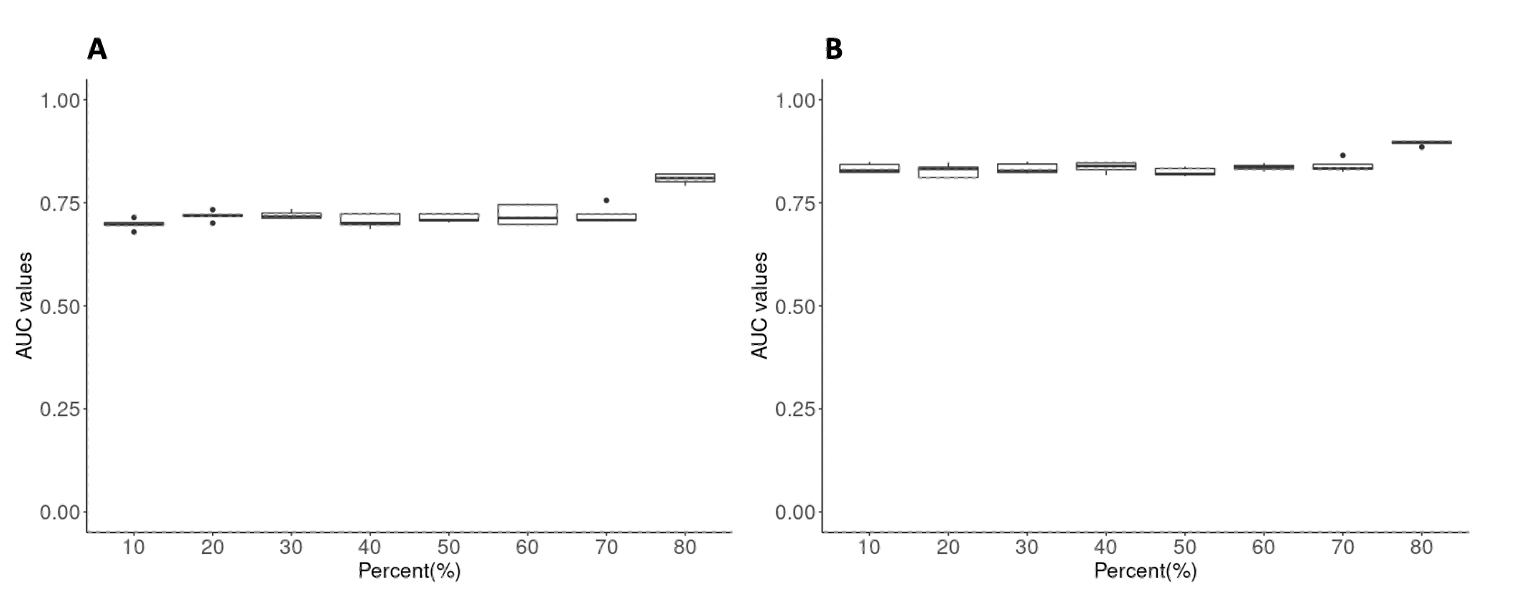

Supplement: S7 Fig — (A) The mononucleotide STR model. (B) The polynucleotide STR model. (TIF) [file pgen.1009604.s007.tif]

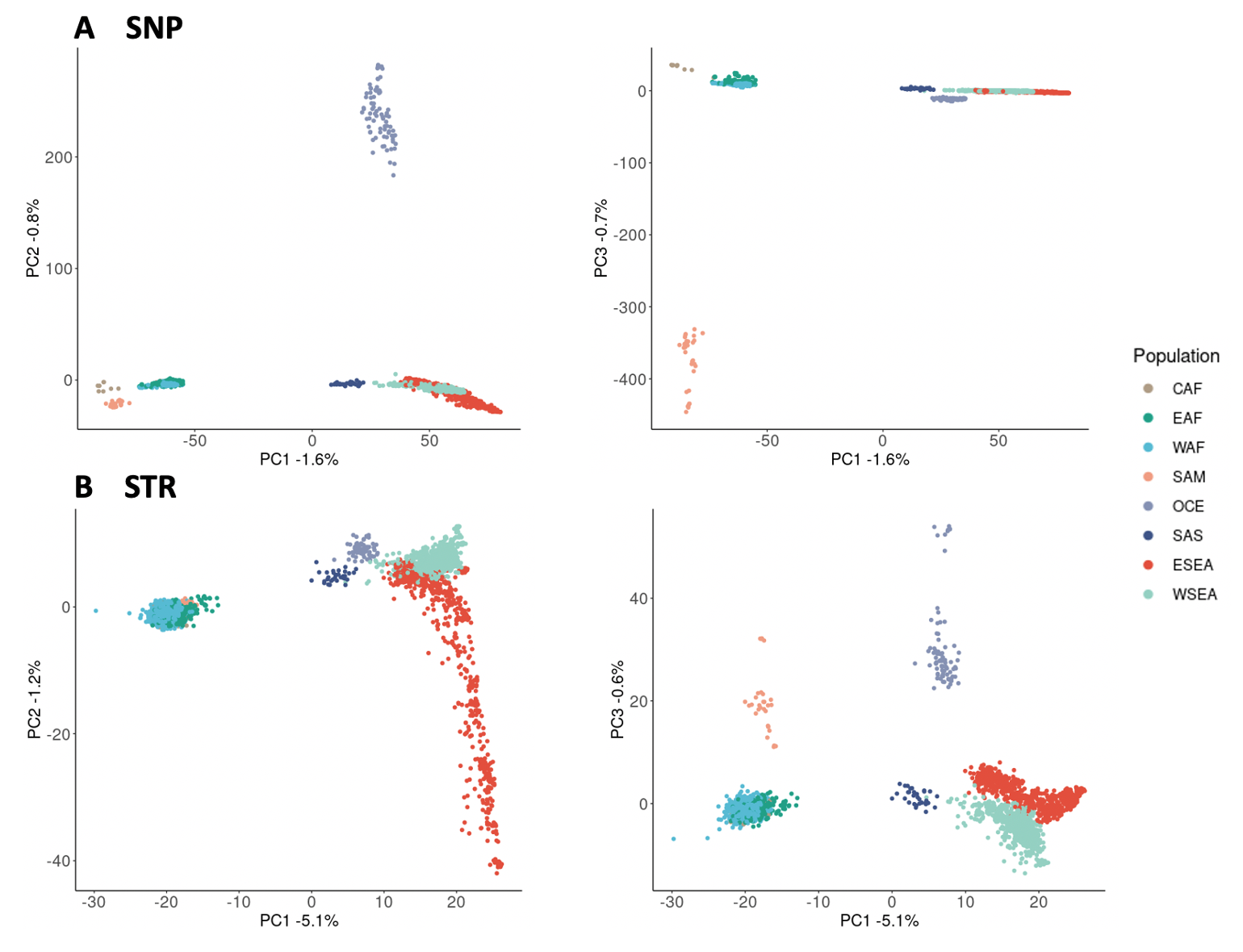

Supplement: S8 Fig — (A) SNP-based PCA based on 213,757 loci. (B) STR-based PCA based on 6,768 (2,563 mononucleotide STR and 4,205 polynucleotide STR) high-quality loci. (TIF) [file pgen.1009604.s008.tif]

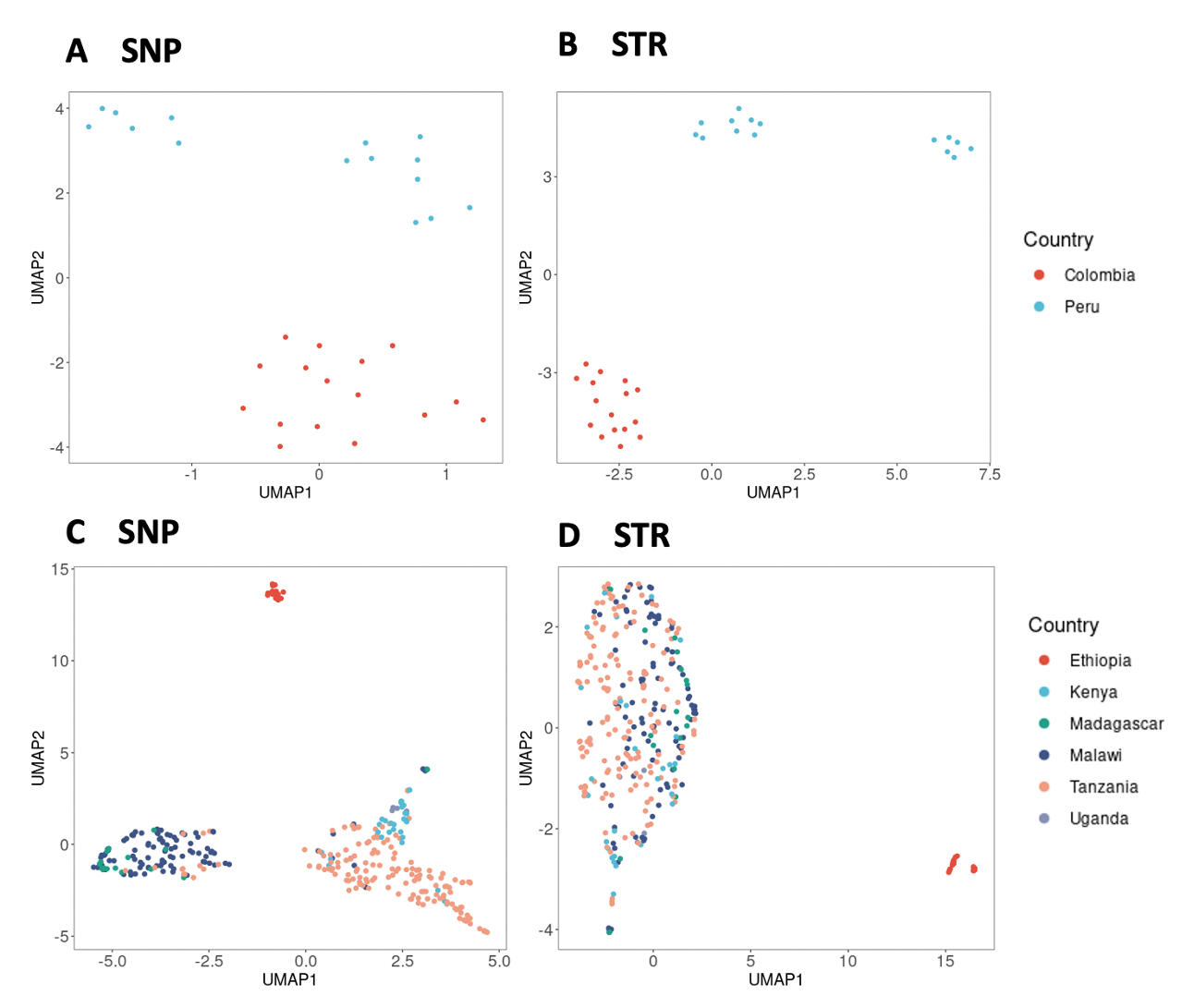

Supplement: S9 Fig — (A) UMAP on the top five principal components (PCs) of the SNP data (SAM countries). (B) UMAP on the top five PCs of the STR data (SAM countries). Colouring the points by the SAM countries. (C) UMAP on the top five PCs of the SNP data (EAF countries). (D) UMAP on the top five PCs of the STR data (EAF countries). Colouring the points by the EAF countries. (TIF) [file pgen.1009604.s009.tif]

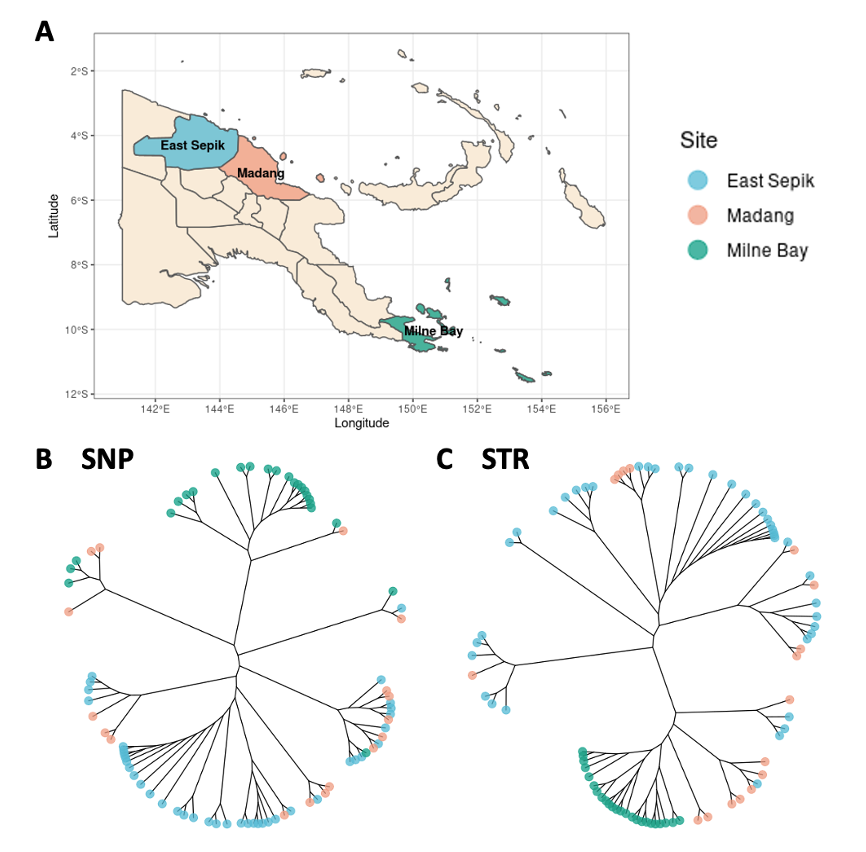

Supplement: S10 Fig — (A) Map showing the geographical location of the three different sample collection sites from PNG. Map drawn with the data from Natural Earth (http://www.naturalearthdata.com/) by the R package rnaturalearth (Version 0.1.0) (https://github.com/ropensci/rnaturalearth) under a CC BY license. (B) Neighbor joining tree based on the SNP data. (C) Neighbor joining tree based on the STR data. Branches are colored according to the site. (TIF) [file pgen.1009604.s010.tif]

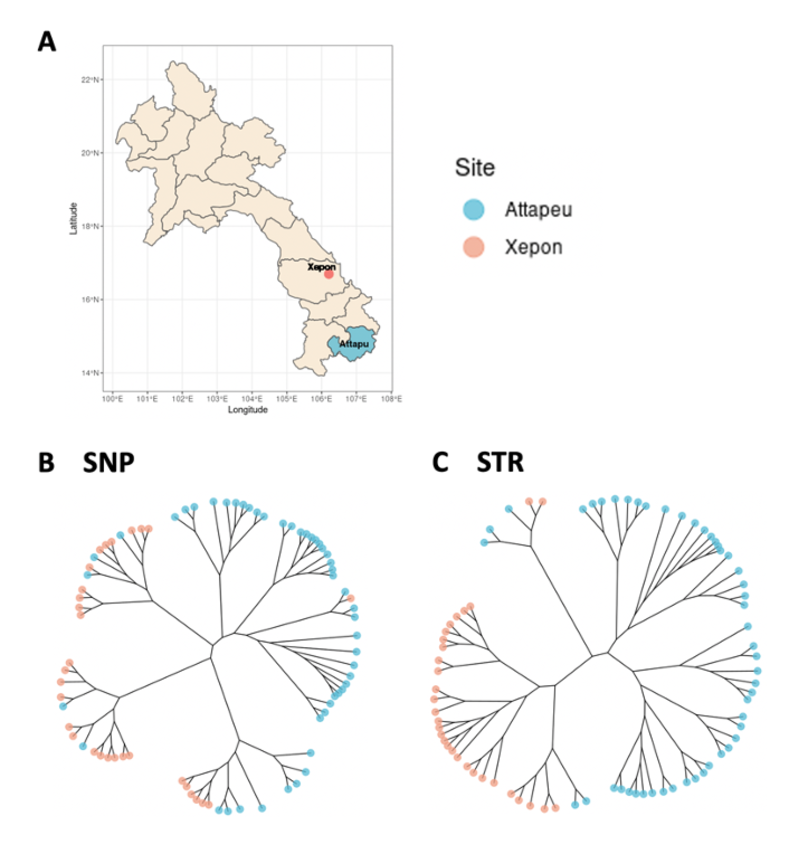

Supplement: S11 Fig — (A) Map showing the geographical location of the two different sample collection sites from Laos. Map drawn with the data from Natural Earth (http://www.naturalearthdata.com/) by the R package rnaturalearth (Version 0.1.0) (https://github.com/ropensci/rnaturalearth) under a CC BY license. (B) Neighbor joining tree based on the SNP data. (C) Neighbor joining tree based on the STR data. Branches are colored according to the site. (TIF) [file pgen.1009604.s011.tif]

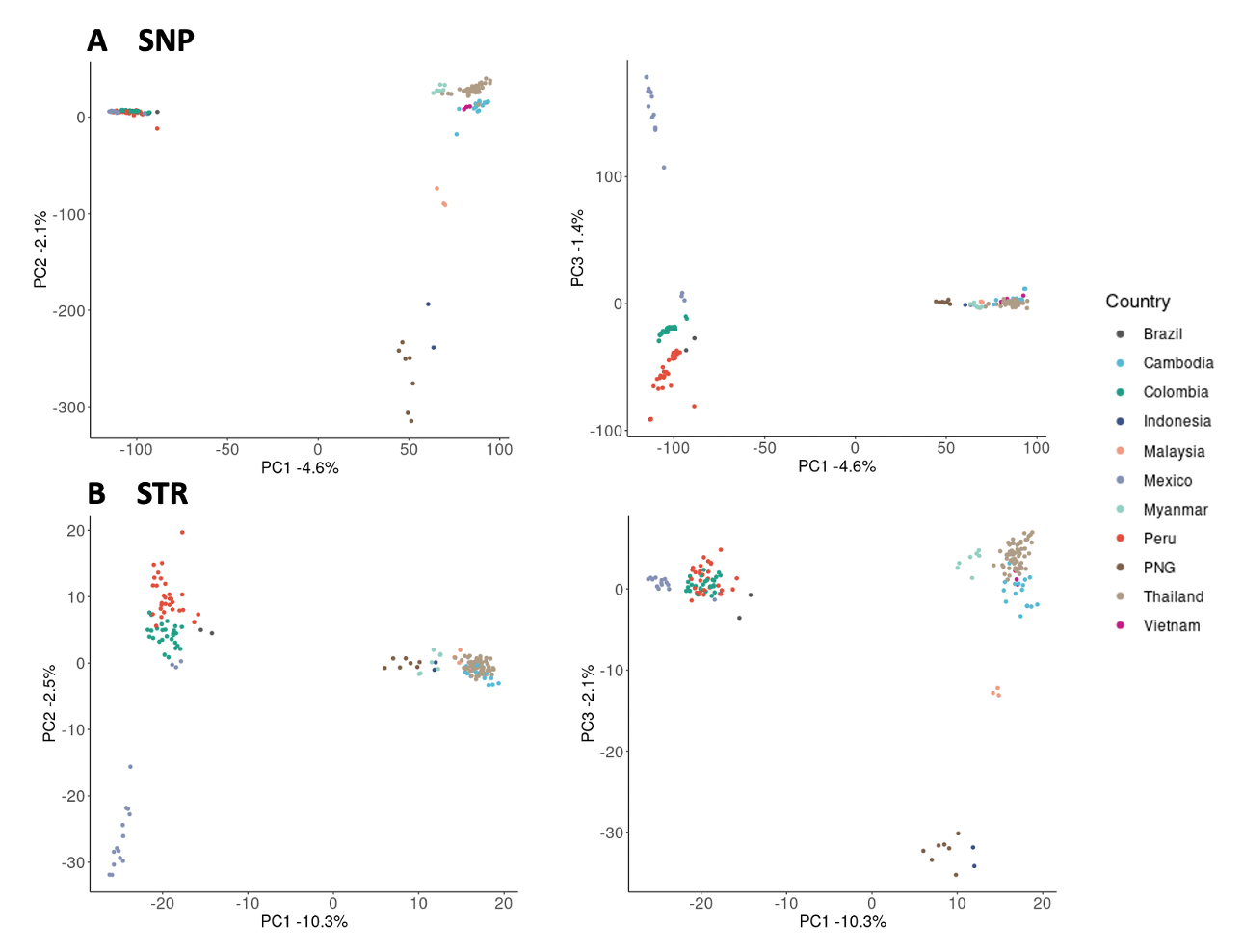

Supplement: S12 Fig — (A) SNP-based PCA based on 188,571 loci. (B) STR-based PCA based on 3,496 (1,648 mononucleotide STR and 1,848 polynucleotide STR) high-quality loci. (TIF) [file pgen.1009604.s012.tif]

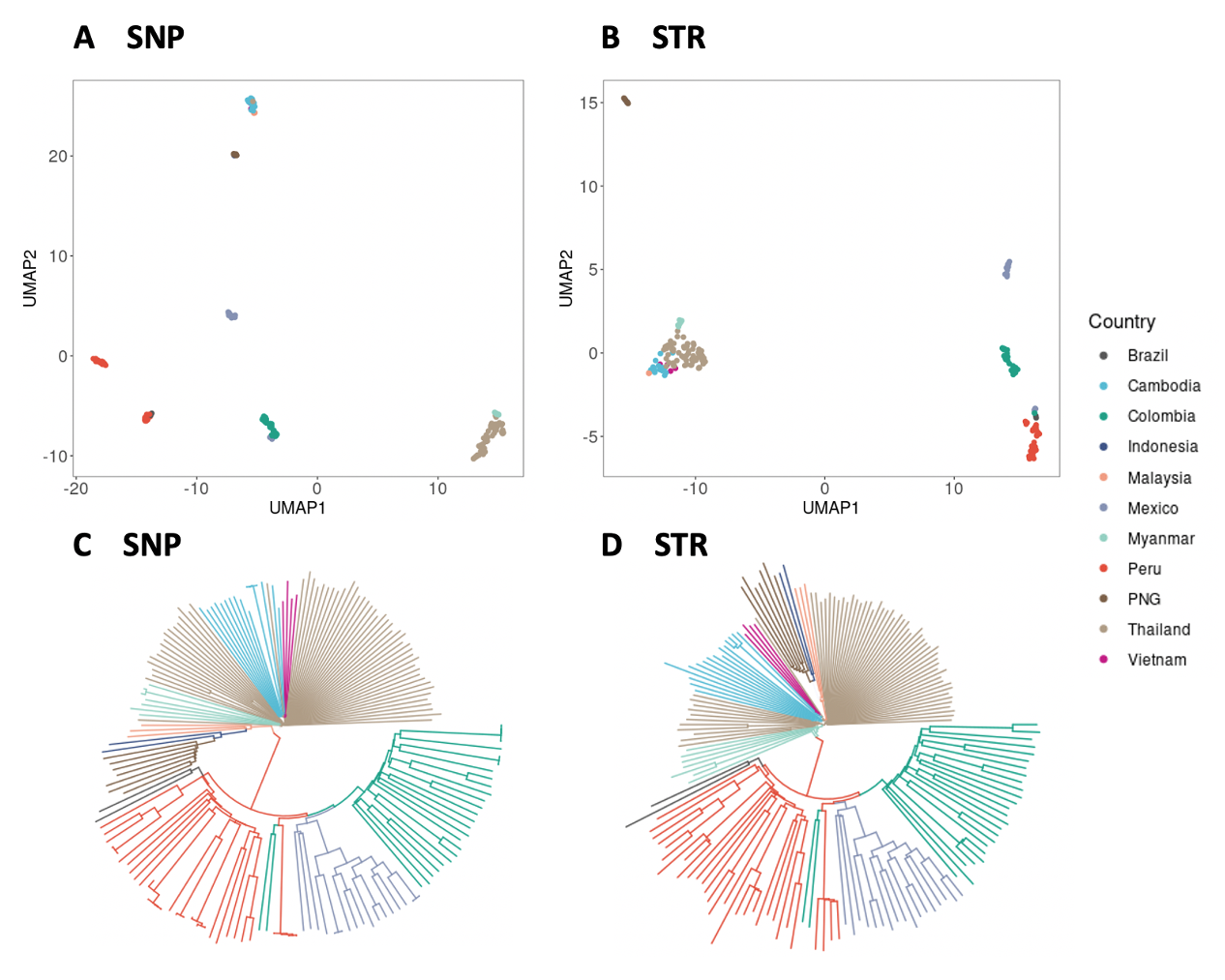

Supplement: S13 Fig — (A) UMAP clustering of the top five PCs of the SNP data with different colors representing the 11 different countries. (B) UMAP clustering of the top five PCs of the STR data. (C) Neighbor joining tree based on the SNP data. (D) Neighbor joining tree based on the STR data. Branches are colored according to the country. (TIF) [file pgen.1009604.s013.tif]

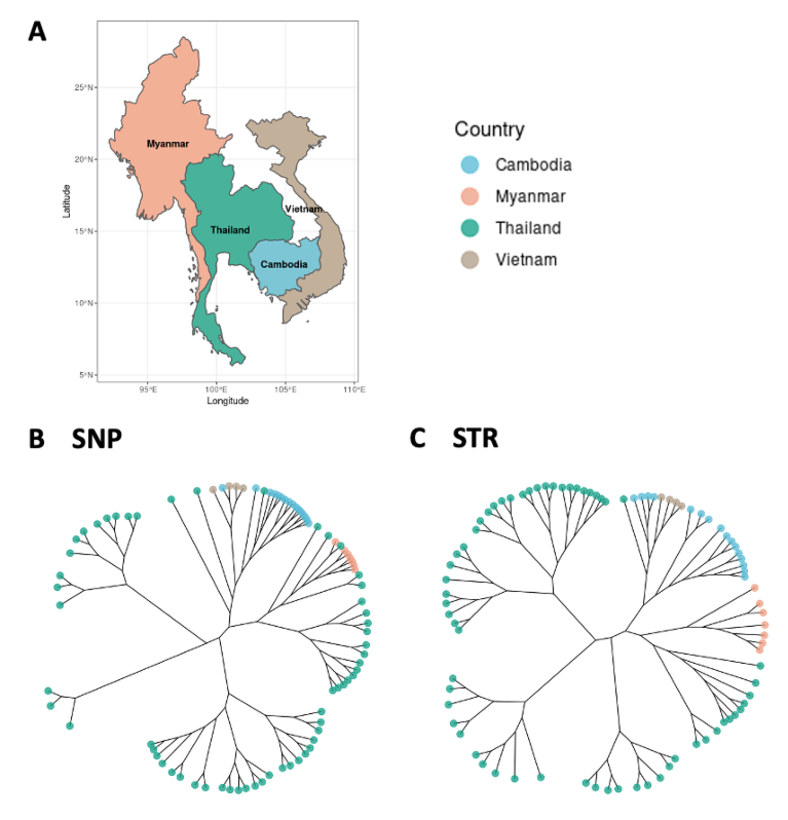

Supplement: S14 Fig — (A) Map showing the geographical location of the four countries. Map drawn with the data from Natural Earth (http://www.naturalearthdata.com/) by the R package rnaturalearth (Version 0.1.0) (https://github.com/ropensci/rnaturalearth) under a CC BY license. (B) Neighbor joining tree based on the SNP data. (C) Neighbor joining tree based on the STR data. Branches are colored according to the country. (TIF) [file pgen.1009604.s014.tif]

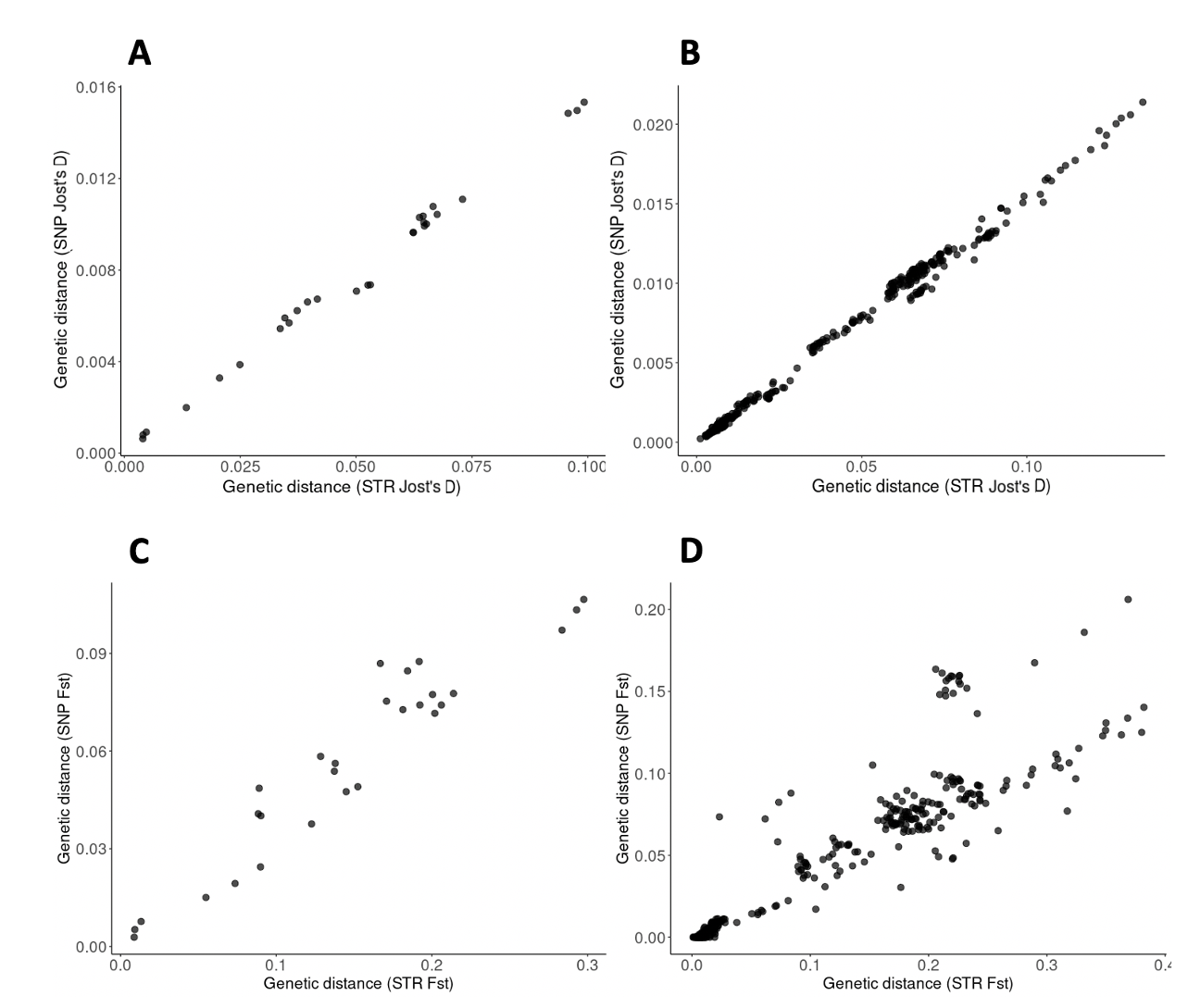

Supplement: S15 Fig — (A) Jost’s D of population pairs (Mantel r = 0.996, P = 0.001). (B) Jost’s D of country pairs (Mantel r = 0.996, P = 0.001). (C) FST of population pairs (Mantel r = 0.97, P = 0.001). (D) FST of country pairs (Mantel r = 0.90, P = 0.001). Mantel tests were used to measure the correlation. (TIF) [file pgen.1009604.s015.tif]

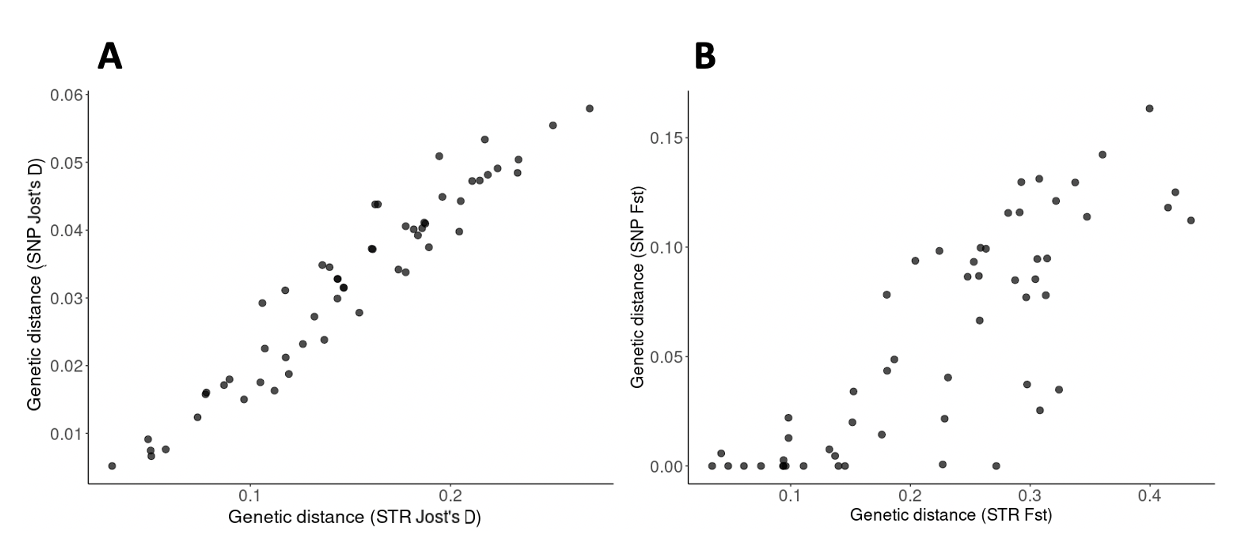

Supplement: S16 Fig — (A) Jost’s D of country pairs (Mantel r = 0.9678, P = 0.001). (B) FST of country pairs (Mantel r = 0.9185, P = 0.001). Mantel tests were used to measure the correlation. (TIF) [file pgen.1009604.s016.tif]

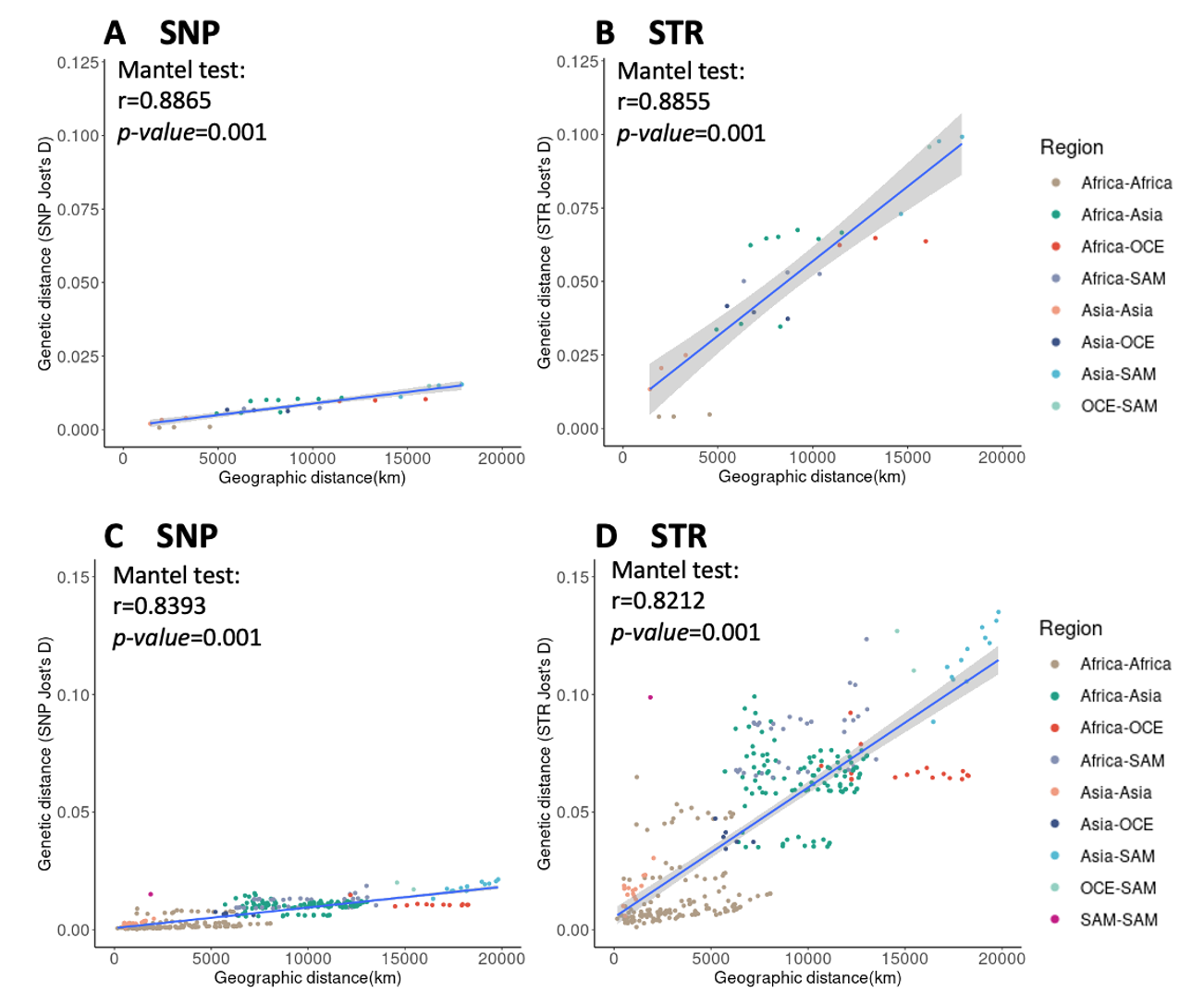

Supplement: S17 Fig — (A) SNP data of population pairs. (B) STR data of population pairs. (C) SNP data of country pairs. (D) STR data of country pairs. A Mantel test was used to measure the association. (TIF) [file pgen.1009604.s017.tif]

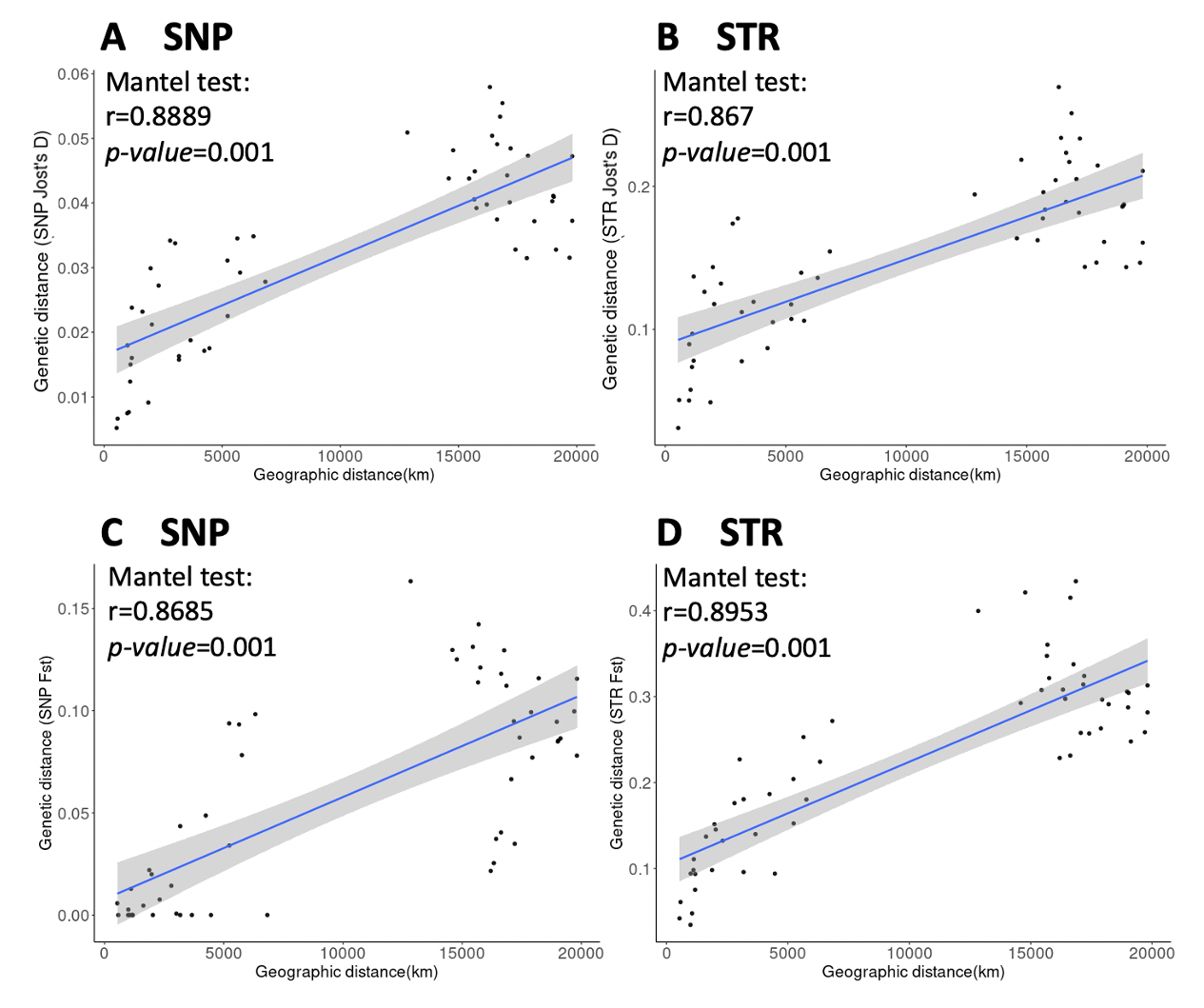

Supplement: S18 Fig — (A) SNP data of country pairs (Jost’s D). (B) STR data of country pairs (Jost’s D). (C) SNP data of country pairs (FST). (D) STR data of country pairs (FST). A Mantel test was used to measure the association. (TIF) [file pgen.1009604.s018.tif]

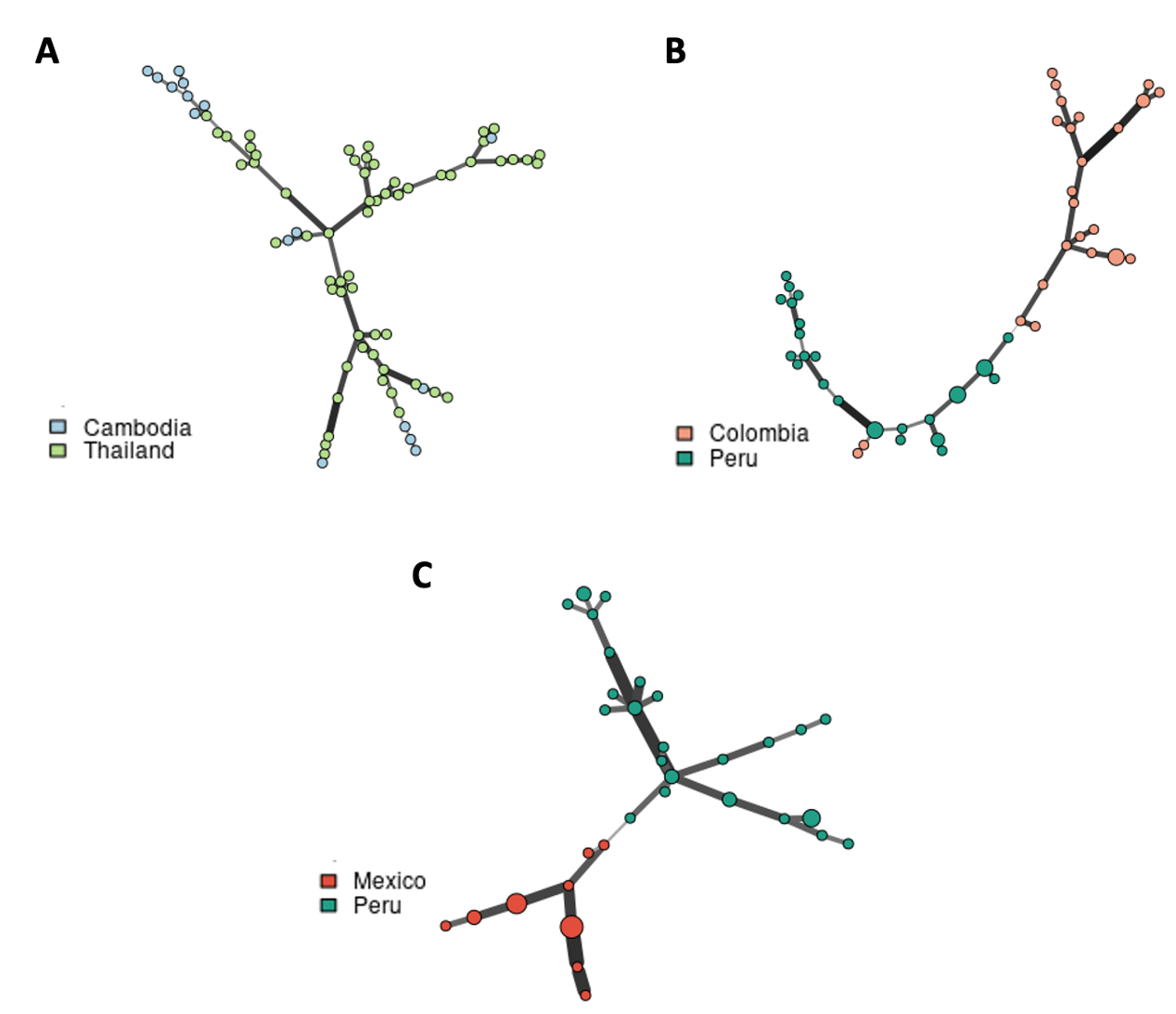

Supplement: S19 Fig — (A) Cambodia and Thailand. (B) Colombia and Peru. (C) Mexico and Peru. Colors correspond to the country. Node sizes correspond to the number of samples. Edge lengths are arbitrary. (TIF) [file pgen.1009604.s019.tif]

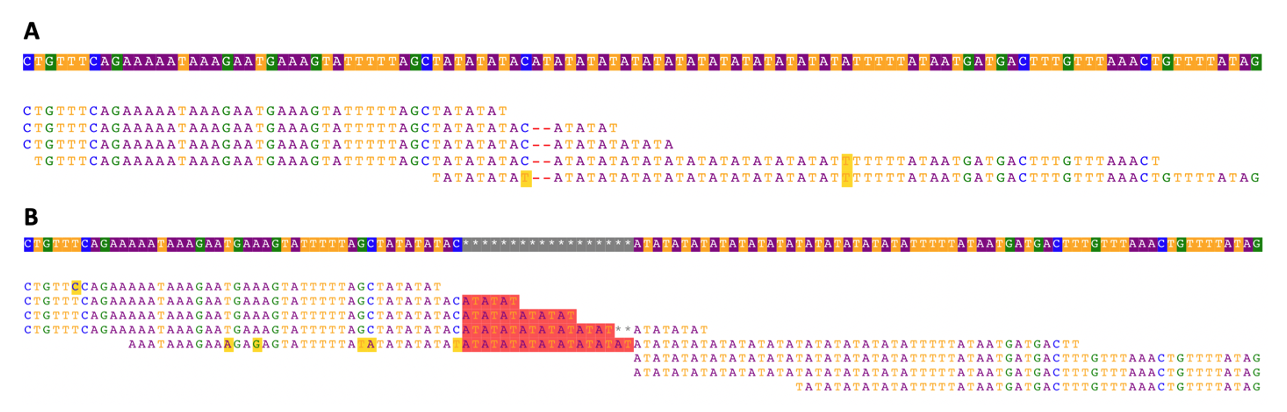

Supplement: S20 Fig — (A) One drug-resistant sample. (B) One drug-sensitive sample. (TIF) [file pgen.1009604.s020.tif]
