# Supplementary material for: Population-level genome-wide STR discovery and validation for population structure and genetic diversity assessment of Plasmodium species
Source: PLoS Genet. 2022 Jan 10;18(1):e1009604. doi: 10.1371/journal.pgen.1009604 (PMC8782505; doi:10.1371/journal.pgen.1009604)
Supplement: Attachment — Submitted filename: Response to reviewers.pdf [file pgen.1009604.s034.pdf]

We thank all of the three reviewers for their suggestions and we address all of their queries below, point by point.

Reviewer 1.

The manuscript by Han et al, describes tools for exploring and identifying STRs in the genome of P. falciparum and P. vivax. As has been the case for other species, methods to identify and filter SNPs have well outpaced STRs for understand malaria population genetics. To this end the authors propose a regression based approach to aid in identifying genuine variation. While the area of STR typing is of high interest to malaria researchers, there are concerns I have on the novelty, validation and generalizability of the paper. Overall, I wanted to see a greater investment in showing how STRs may be superior to SNPs to understand structure and selection in the parasite genome, especially over timescale where the higher mutability of STRs adds critical detail. STRs could be a really powerful alternative to SNPs, I would love to see this demonstrated more clearly.

1. Novelty:

(a) The authors state that this is the "first large-scale STR typing study". I disagree with this characterization. The data in the study is a subset of 7,000 samples published by the MalariaGEN consortium. A paper cited in the current manuscript (Ahoidi et al, Wellcome Open Research) reports ~3,000,000 indels typed across the 7,000 samples. STRs clearly compose a subset of these.

We thank the reviewer for this comment. The paper (Ahouidi *et al*. 2021) used the GATK HaplotypeCaller which identified a total of 1,838,733 SNPs (of which 1,626,886 were biallelic) and 1,276,027 indels (or SNP/indel combinations). The population genetic analyses in this paper only focus on high-quality SNP genotypes, ignoring the indels which contain a small subset of STRs.

Insertions/deletions (indels) and STR whilst sharing some overlap (small STR size changes may be called as indels) are not capturing the same information. Most indels are similar to SNPs in that they are typically biallelic, of smaller size, and have a lower mutation rate than STRs. We argue that using a specific STR caller like HipSTR reveals far more information for STRs than using an SNP/indel caller, such as GATK HaplotypeCaller (Halman *et al*. 2020). We have updated our introduction to make this point (Line 101).

(b) In addition, the authors cite several other papers (including some which also use HipSTR) which report significantly sized data (albeit not in the thousands).

We have inserted sentences (Line 91, Line 95) in the introduction to highlight the orders of magnitude differences in sample size of these previous studies in comparison to our study and to also highlight that these are performed on laboratory-adapted parasite clones, not field samples, which is the focus of our paper. The latter is a much more challenging proposition in terms of data analysis but also with substantial gains in understanding of STR population genetics in the largest population-based *Plasmodium* cohorts analysed to date for STRs.

(c) One omission is the work of Redmond et al (MBE May 2018) where a careful treatment of InDels/STRs is made using PCR-free libraries and far longer reads (250bp) more effectively controlling error rates.

We thank the reviewer for bringing this to our attention and agree that the PCR-free Illumina library with longer reads (250bp) can improve the STR genotyping accuracy. The short-read lengths cannot span large STR loci which means that the set of STRs revealed in this study is still incomplete, but we argue that we have captured the vast majority of relevant STRs. In our analyses, we only considered STRs shorter than 70bp, as genotyping STRs that exceed 70bp invariably requires longer reads (Willems *et al*. 2017), but most *Plasmodium* STRs are much shorter than this threshold. We identified 105,844 STRs from the *P. falciparum* Pf3D7 reference genome (104,649 STRs shorter than 70 bp, accounting for 98.87%) and 40,468 STRs from the *P. vivax* PvP01 reference genome (40,224 STRs shorter than 70 bp, accounting for 99.40%). Overall, the short Illumina reads are sufficient to characterize the majority of STRs in the *Plasmodium* genome. We have updated our discussion to make this point more clearly and to outline the limitations due to the short reads (Line 564).

(d) Several of the cited works also use crafty validation approaches (inheritance in genetic crosses/augmentation of reference sequences) which could inform the current work.

We thank the reviewer for this suggestion and have now included the Pf3K genetic crosses data (https://www.malariagen.net/parasite/p-falciparum-genetic-crosses) performing extensive analyses of this newly added dataset as part of this revision.

(e) Some chunk of the paper has been devoted to the distribution of STRs through the genome. To some extent this is a retreading of other papers (notably Hamilton et al and McDew-White et al).

We thank the reviewer for highlighting this and we have now revised this part accordingly (Line 161; 168).

2. Validation:
(a) The comparison to the gel electrophoresis is a great idea, though oddly no estimate of the accuracy was determined in this manner. The data looks supportive – though it is difficult to visually interpret the point sizes. Can the numbers here be made explicit and used to derived a measure of accuracy. To have this as the sole direct measure of accuracy is disappointing. There are manifold ways to derive this – as above, either using mendelian error rates from genetic crosses, augmenting reference sequences, or looking at concordance between technical replicates would all be valuable. Technical reps are gathered, it is not clear how these have been leveraged.

We thank the reviewer for this great suggestion and have undertaken two additional pieces of work:

1) Capillary electrophoresis data is typically taken to be the gold standard for STR genotyping (Willems *et al*. 2017), although its use as a gold standard is debatable due to a number of underlying issues, especially when multiple clones are present in malaria samples (Anderson *et al.*

1999; Schultz *et al*. 2010). We have provided the source allele length (bp) of *P. falciparum* STR HipSTR calls and gel electrophoresis (GE) data (S1 Table) and added the R-squared (R^2) value to the supplementary figure (S4 Fig).
2) We have now included the Pf3K genetic cross dataset as a new data analysis. We have now included these results, which support our developed filtering strategy in the manuscript main text (Line 172) and the methods section (Line 643). We have also added figures and tables to the supplementary data (S1 Fig; S2 Fig; S3 Fig; S8 Table; S9 Table; S10 Table and S11 Table) displaying the accuracy of the HipSTR calls before and after our filtering approach as well as compared to the SNP genotyping by comparing the Mendelian error rate within the crosses and genotype discordance across replicates.

(b) I have significant issues with the regression based approach. To my reading, STR quality was determined by how well an STRs genotype conformed to an estimate of population structure derived from SNPs (either the first 5 or 10 PCs, depending on the text vs. methods). STR quality is not really defined clearly, though I have a fundamental issue (or a fundamental misunderstanding) of how this approach works. Firstly, I would not expect any specific STR to match the global population structure from SNPs. The STRs reflect the local structure at that point along the chromosome. The local structure would not necessarily reflect the global (for instance in the case of selective sweeps surrounding drug resistance genes), and even locally the mutability of STRs would suggest that the impact of sweeps would be retained over different timescales than for SNPs so that local structure would not necessarily be maintained.

We thank the reviewer for this comment.

STRs have been used for decades, extensively, for association and linkage studies across many species, in addition to population studies. Although having a higher mutation rate than SNPs it is acknowledged that STR and SNPs should overlap to a large extent on their inference of broad geographic/population-level signals (Bradbury *et al*. 2018; Sunde *et al*. 2020).

One of the key problems for the application of in silico STR genotyping using tools such as HipSTR in population cohorts of field samples (without the availability of technical replicates) is the lack of assurance regarding the quality of the genotype calls.

Our solution to this problem is to utilise the fact that STRs not only reflect local SNPs but also that they will reflect global population structure and that this should, to a large extent, mirror population structure as inferred by SNPs, with some added resolution for more recently evolved population structure due to the higher mutation rate. It is the primary premise of our method that we sought to exploit with our principal component/regression based approach. We hypothesised that this approach would lead to a useful novel filtering strategy that can identify high fidelity STR markers based on in-silico algorithms which could be applied in any species.

(c) The model is split by mono and polynucleotide repeats. However, particularly for falciparum, there are almost no 2bp repeats in coding regions, and almost no 3bp repeats outside of them. Sequence quality varies considerably between coding and non-coding regions. How is this accounted for?

We also observed the well known GC-content difference in *Plasmodium falciparum* with the AT-content of the *P. falciparum* genome within coding sequences estimated as ~75%, while in non-coding regions the AT-content rises to ~ 90–95%. Our regression model accounts for these abrupt changes in GC content in three ways: (i) by considering the GC-content of the STR repeat itself, (ii) as well as the GC-content of the region around each STR locus, and (iii) the contrast in GC-content of the repeat motif and its surrounding sequence in the model. We observed that the *P. falciparum* model showed GC_Diff and GC_Flank variables to have a positive correlation with STR quality (Table 1) and thus these terms were included in our filtering strategy.

(d) A goal of the paper is to capture differences between SNPs and STRs for population structure, if there is a filtering of STRs to only retain those which reflect structure from SNPs then there seems little value in using them instead of SNPs. The regression model appears to be validated based upon how closely the STRs match the population structure. It would appear to be trained to find a set of markers which match the population structure defined by SNPs rather than to find accurately called STRs.

We agree with the reviewer's comment that there is some circularity in these results since our QC measure was predicated on capturing geographic information. However, when building the multivariable logistic regression model, the outcome of high-quality or low-quality STRs is based on different SNP PCs defined *a priori*. Then multiple independent variables (S2 Table) were selected based on the STR feature and used for the prediction of the STR quality. After building the model, we set the cut-off for predicted probabilities to distinguish high-quality or low-quality STRs evaluated by calculating the area-under-the-curve (AUC) on the receiver–operator–characteristic (ROC) curve. Therefore, these selected STRs are not only related to SNP PCs but also keep STR features. We validated the approach with cross fold validation and found it to be robust.

(e) I also find it odd that expected heterozygosity and Jost's D are in the model. Surely, these should be independent of the model so that signal of selection are not driven by population structure?

We thank the reviewer for this comment. For genetic diversity (e.g., expected heterozygosity and Jost's D), we calculated this for each STR locus. These represent the STR-specific genetic information and are independent variables. These variables are independently associated with the outcome of the R^2 correlation coefficient that we defined based on SNP PCs (they retain their significant p-value in the model despite the inclusion of other variables). As such, we feel that is valid and important to keep these variables in the model.

3. Selection:

(a) The scans for selection for drug resistance genes lacks a clear rationale. EAF samples are split by the presence of K76T at pfcrt. Correspondingly, divergence around this allele is found, presumably due to the global selective sweep at this locus. Why would any other loci show significant divergence? Chloroquine has not been used for some time in east Africa and any selection maintaining interchromosomal linkage is likely diminished.

We thank the reviewer for this comment. The rationale here is to screen the STRs that might represent the selection signature related to drug resistance by performing a genome-wide scan among the drug-resistant and drug-sensitive samples. After comparing chloroquine 57 EAF drug-resistant and 269 EAF drug-sensitive samples, we found that some STR loci near *Pfcrt* showed significant divergence, which might be due to linkage disequilibrium (LD) surrounding the *Pfcrt* gene due to selective sweeps. Previous studies revealed the STR markers linked with known drug resistance gene might have strong directional selective sweeps and show extensive LD surrounding the known drug resistance gene, e.g., *Pfcrt* (Wootton *et al*. 2002), *Pfdhfr* and *Pfdhps* (McCollum *et al*. 2008; McCollum *et al*. 2012). However, some other loci also showed significant divergence, the reason might be that the STR loci reflect the geographical differentiation of the isolates. Most drug-resistant samples from EAF are from Ethiopia, and previous studies have shown that Ethiopia is a distinct subpopulation from the rest of Africa (Amambua-Ngwa *et al*. 2019; Ahouidi *et al*. 2021). Lastly, it is our understanding that several of the sub cohorts included in the MalariaGEN dataset were collected many years ago and do still reflect the chloroquine selection signature. Indeed, our previous work on IBD selection signatures in the MalariaGEN consortium Pf3k project dataset clearly identified the chloroquine signature (with SNP data) surrounding *Pfcrt* as well as the loci in other chromosomes in multiple subpopulations such as Ghana, Guinea, Mali (Henden *et al,* PLOS Genetics 2018).

(b) For the analysis in SE Asia, this is again quite confusing. Artemisinin resistance has arisen on hundreds of different genetic backgrounds, it is very challenging to understand what these other loci mean. Notably, they appear all reflect differences in historical drug use between Laos and Thailand, rather than being involved in artemisinin resistance. For instance, the chr 5 is near pfmdr1, the chr 12 locus is near GTP-cyclohydrolase suggesting the impact of mefloquine/anti-folate use.

We thank the reviewer for this comment and agree the analysis in SE Asia is not very compelling. Many STRs showed a significant divergence between Laos and Thailand, which might be reflected in differences in historical drug use and not only related to artemisinin. The complex genetic backgrounds of artemisinin resistance in SE Asia and the small sample size in Thailand (16) make the results less powerful and difficult to determine. As such, we have edited the manuscript and deleted the analysis in SE Asia. The focus of the manuscript is the utility of in silico STRs genotyping in malaria field samples.

(c) Were attempts made to use alternative selection statistics, for instance those which use haplotype information. A highly mutable marker would seem to offer a very low background signal for such approaches.

We thank the reviewer for this suggestion and agree that this is worthwhile but beyond the scope of the paper. In our paper we do compare our results to the results from a previous study (Henden *et al*. 2018) (Line 471). Here we calculated the proportion of pairs identity-by-descent (IBD) at genomic locations across the genome using a multi-SNP technique (a hidden Markov model). We used the IBD signal to identify positively selected regions. This method identified several IBD signals reflecting signatures of positive selection and haplotype differentiation in *P. falciparum* across populations, recapitulating well known signals such as chloroquine selection.

Other methods to detect selection are based on haplotype based tests, such as integrated haplotype score (iHS) (Sabeti *et al*. 2007) and have been extensively applied to SNP based data. We elected not to apply these methods as we feel that their application will require substantial testing and validation when applied to STR data as these methods were designed for SNP markers, which is beyond the scope of this paper. We agree that they would be of interest to apply in the future. It is important to note that STRs have lower linkage disequilibrium, leading to less power for haplotype (and multimarker IBD) tests. On the positive side most STRs have higher information content than any single SNP.

(d) For figure 4 each y-axis is to a different height making it difficult to compare across plots.

We thank the reviewer for this suggestion and we now use the same y-axis in Figure 4 (Line 367).

(f) I was also confused why FWS filtering only was performed on vivax samples

We thank the reviewer for highlighting this. We only used monoclonal samples for both *P. falciparum* and *P. vivax* samples. However, for the *P. falciparum* samples, we downloaded the metadata file from MalariaGEN (https://www.malariagen.net/data/catalogue-genetic-variation-p-falciparum-v6.0)(Line 589). The metadata file included a variable "Measure of complexity of infections" which provided the characterisation of within-host diversity (F_{ws}) for 5,970 QC pass *Plasmodium falciparum* samples which we utilised in our paper. Hence the QC pass had filtered for monoclonality based on F_{ws}. We have now also calculated the F_{ws} of the *P. falciparum* samples and confirmed that they are monoclonal samples (Line 125, Line 682).

Reviewer 2.

This manuscript from Han and colleagues describes the application of a microsatellite genotyping approach (HipSTR) to a large collection of Plasmodium falciparum and Plasmodium vivax malaria parasite short read whole genome sequencing datasets. Though microsats are common in these genomes, they have historically been paid less attention than SNPs due to difficulty in accurately calling microsat length polymorphisms from microsatellite loci. The authors demonstrate that HipSTR microsatellite calls are generally reliable and recapitulate or complement many selection and population structure patterns observed with SNPs. This manuscript represents a solid body of work, and many in the malaria field will be appreciative to see a thoroughly-explored methodology for calling microsat variants in Plasmodium. However, the manuscript could be improved through attention to a few issues:

1) Line85: the authors mention a host of methods for calling microsat/STR variants, but only evaluate HipSTR in this manuscript. Can readers be confident that this is indeed the best tool for Plasmodium? Did the authors evaluate other approaches?

We decided to focus on HipSTR (Willems *et al*. 2017) for several reasons discussed in the introduction (Line 104). Most currently available in silico STR tools were designed to identify the true length of repeats independently in each sample along the genome, while HipSTR considers the entire sequence across all samples in the dataset for each STR site, which reduces the alignment errors, PCR stutter errors, and can obtain more reliable STR genotypes, even with low coverage WGS samples. Thus, HipSTR is the logical choice for large population samples such as MalariaGEN. HipSTR has been shown to perform very well in comparison studies (Willems *et al*.

2017; Gymrek *et al*. 2017; Halman *et al*. 2020) and is one of the most widely used STR callers. We note that our approach is general and could be implemented for other STR genotype callers.

2) The Pf3K dataset includes a set of sequenced progeny from Plasmodium sexual crosses, which were very useful for identifying a gold standard set of variants for developing best practices for SNP calling with GATK. Did the authors consider using this approach for evaluating HipSTR calls, which could offer a more comprehensive validation than gel electrophoresis of a small panel of microsats as in Fig S1? Genome wide selection scans and analyses of population structure are not dependent on uniformly high accuracy of calls, but other applications could require highly accurate calls. Similar to how the Pf3K consortium delimited the 'accessible' regions of th P. falciparum genome for read alignment and accurate SNP calling, it could be very useful to the malaria field to produce a set of coordinates of microsats that would be expected to yield highly accurate calls based on their features and performance with cross progeny.

We thank the reviewer for highlighting this and agree that the Pf3K dataset is a very useful dataset to evaluate the HipSTR calls. We have now included the Pf3K genetic crosses dataset, the parents and 92 progeny clones from three crosses of *P. falciparum* (3D7×HB3, HB3×Dd2, and 7G8×GB4 https://www.malariagen.net/parasite/p-falciparum-genetic-crosses) to evaluate HipSTR calls. We have now included these results, which support our developed filtering strategy in the manuscript main text (Line 172) and the methods section (Line 643). We have also added figures and tables to the supplementary data (S1 Fig; S2 Fig; S3 Fig; S8 Table; S9 Table; S10 Table and S11 Table) displaying the accuracy of the HipSTR calls before and after our filtering approach as well as compared to SNP genotyping by comparing Mendelian error rate within the crosses and genotype discordance across replicates.

We have now also provided the genome-wide accessible STR variation in *P. falciparum* in Rshiny (PlasmoSTR https://github.com/bahlolab/PlasmoSTR) based on *P. falciparum* genetic crosses data (https://www.malariagen.net/parasite/p-falciparum-genetic-crosses). These STRs show highly accurate calls (No Mendelian errors or discordance between biological replicates) in three experimental genetic crosses of *P. falciparum* (98 samples).

3) Line 270 and Figure 3: I don't understand what is meant by the claim that STRs provide 'greater resolution of distinct samples at the local scale' than SNPs, considering that the analyzed samples do not come up local-scale geographic metadata below the level of country. The SNP vs. STR plots in Figure 3 look generally similar, and without metadata indicating the truth about precisely when and where samples were collected, how is it possible to claim that one UMAP plot or NJ tree provides more resolution than another?

We thank the reviewer for this comment and agree that this sentence is not accurate as it is only visible/noticeable at the "Country" level. The *P. falciparum* samples contains the "Site" information (below the level of the "Country") in the metadata, we then explored the local levels of parasite population structure and found at some local sites, the STR data formed a well-separated and distinct cluster with the different sites, while SNP data was unable to separate these sites into distinct clusters demonstrating that STRs can identify more recent (often local) stratification, missed by SNP data (Line 327; Line 339; S9 Fig; S10 Fig; S13 Fig).

4) The Discussion section is very long and spends much time simply recapitulating the Results. It would be helpful to better contextualize key results in this section. This manuscript generally shows that microsats/STRs can recapitulate many findings first observed with SNPs in P. falciparum or P. vivax. Can the authors further comment on why microsats should be profiled on a genome-wide scale? Some purported advantages of microsats over SNPs are not clear. For example, (line 559) why would microsats reveal recent signals of selection more quickly than SNPs? Their capacity for recurrent mutation would seem to make them less useful in general for tagging genomic variants under selection, and reason suggests that strong directional selection would result in sweep signals simultaneously in SNP and microsatellite markers.

We thank the reviewer for this suggestion and have made efforts to make our discussion more informative as well as reducing the text that recapitulated the results.

Small points:

1) Figure 3 caption, part B is a trailing sentence.

We thank the reviewer for noticing this. We have now made changes to the Figure 3 caption (Line 321).

<u>Reviewer 3.</u>

The authors present interesting and well-done work to use short tandem repeat (STR) markers for population genetic analysis, the first large-scale application in malaria parasites. Their approach is thorough: using cutting edge bioinformatics tools, developing a set of multi variate regression models to predict STR quality, wet-lab crosschecks of their predictions a detailed direct comparison to the standard SNP-based methodologies for a range of population parameters. Their write-up is comprehensive and interesting, providing strong context useful for a general audience and citations. It is impressive that the authors have generated a web-based R Shiny application, PlasmoSTR and make extensive supporting information available.

STRs have many appealing features for genetic analysis but they have not been readily accessible to users due to the many challenges of assaying them accurately, lack of quality controls and a general unknowns with respect to their performance. The authors have gone to great effort to build a tool that is easily accessible and user-friendly. They then applied it to a massive dataset (3K *P. falciparum*, 174 *P. vivax*) whole genome sequences. It is likely that this baseline information will be heavily used/cited not only by malaria researchers but by those who would emulate this same optimization template for their own species.

In terms of biological discovery and novel knowledge gained, this work as presented is less forceful, not as coherent and generally lacks a strong take-home message. The authors can do more to make these points and argue more effectively that STRs in malaria parasites can be a game-changer, particularly by being more detailed about what the particular strengths and weaknesses of this approach are; there are a wide range of variables and parameters that are only cursorily considered but are very important.

Detailed comments:

1. The title states "higher resolution population structure". There are some examples in the data that support this explicitly, but in general the data are more nuanced. For some kinds of questions STRs may offer an advantage over SNP-based methods. It would strengthen the value of this work to specify more clearly what is gained in which circumstances. It seems more likely that STRs can be a useful companion to SNPs.

We thank the reviewer for this suggestion and have made efforts to make the discussion clearer and more informative further highlighting the benefits of STRs.

2. There are a wide range of variables involved that are both fascinating and possibly confounding. The authors do a good job of mentioning them but fall short of making definitive statements in most cases. It would be helpful to the reader to better understand the information content breakdown for specific types of evolutionary questions. Mono vs Poly tracts (and type of poly), length of the STR, position in the genome, position in the gene (if it impacts a gene).

We thank the reviewer for this suggestion. The population genetic analyses in this paper are based on genome-wide high-quality STRs. For this sets of STRs we provided the STR motif type, STR motif copy number, STR Locus (position in the genome), gene description for each STR locus in the Rshiny tool (PlasmoSTR https://github.com/bahlolab/PlasmoSTR). In this revision we have now provided additional information for each STR locus in the Rshiny tool, which now also shows the gene name, gene link to PlasmoDB, AlphaFold (Jumper *et al*. 2021) predictions link for the coding STRs, and also included the STR multivariable logistic regression model parameter (Pf and Pv) in the Rshiny. We hope these additional, searchable results will be useful to the readers.

STR Locus (Pf3D7)	Motif	# copies (Pf3D7)	Gene	Product.Description	Category	Heterozygosity	PlasmoDB_Link	Protein_Link
All	All	All	All	All	All	All	All	All
1:135875-135903	CCTTTT	5	PF3D7_0103100	vacuolar protein sorting-associated protein 51, putative	coding	0.36	PlasmoDB	AlphaFold
1:151634-151656	ATATTT	4	PF3D7_0103400	zinc-carboxypeptidase, putative	coding	0.274	PlasmoDB	AlphaFold
1:155820-155841	AAT	7	PF3D7_0103500	conserved Plasmodium protein, unknown function	coding	0.637	PlasmoDB	AlphaFold

3. Especially with respect to the question of whether the marker itself is under selection would have a big impact on the result and interpretation. A preponderance of the STRs resides in promotors. The authors note that this large sequencing dataset is not accompanied by RNA-seq data. Do they have other ways (other datasets) to ascertain their impact? Indeed, the functional implications of STRs are very interesting on their own, but how that interfaces with being high quality genome-wide markers needs more consideration.

We thank the reviewer for highlighting this and agree that this is a fascinating question and very important to understand the impact of STR variation, but beyond the scope of this manuscript. We also note that this is a difficult question and has only been fleetingly addressed in human (Fotsing *et al*. 2019) and *Arabidopsis thaliana* (Reinar *et al*. 2018) studies.

One recent study (Fotsing *et al*. 2019, same lab that developed HipSTR), performed a genome-wide analysis (expressionSTR or eSTR analysis) to identify the impact of STR variation on gene

expression in humans. They focused on 652 individuals from the GTEx dataset for which both high-coverage WGS and RNA-sequencing data of 17 tissues were available. We have checked the available data in *Plasmodium* and by searching databases and papers and at the moment there is no comparable data available to GTEx in *Plasmodium* and thus we are unable to conduct this *Plasmodium* eSTR analysis. However, this is an important future direction which will help to address functional aspects of STR variation in *Plasmodium* and test whether differences in STR lengths influence gene expression. We have highlighted this point in the discussion (Line 557; Line 561).

4. For genome wide genetic differentiation measures, what does generally "higher values" than SNPs accomplish? Does that indicate better information?

SNP and STR genetic differentiation measures displayed a consistent pattern of which populations were most similar, but STRs provide higher values for these measures, indicating that they had greater dynamic range. This is because each STR locus will almost always have higher per locus allelic diversity than biallelic SNP loci, and therefore result in higher estimates. Compared to the biallelic SNP locus, the STR (multiple alleles) locus has an increased chance to detect the difference between populations, especially closely related populations. We have clarified this statement in the discussion (Line 524).

5. The authors make interesting observations about the value of STRs in local/recent evolution (or maybe better describes as monitoring of genome variants, clonal expansions, recrudescence vs reinfection. These are VERY important for malaria control monitoring and could be more strongly focused on, and in consideration if that might make these markers less useful for deeper evolution studies.

We thank the reviewer for this comment. Our results demonstrated that STRs perform better when determining the genetic structures of individuals at a local scale than SNPs (S9 Fig; S10 Fig; S13 Fig), which suggests that the STRs might be more informative in detecting fine scale differentiation. This, in turn, could help in detecting more recent selection which could be valuable for drug resistance monitoring. Moreover, a higher mutation rate in STRs provides sufficient variation and could be used to distinguish closely related parasites which becomes important when countries are close to elimination and the effective population size is small, making it hard to identify transmission versus recurrence-relapse. Some previous studies used a set of STR markers to identify relationships between primary infection and recurrence-relapse isolates in *P. falciparum* and *P. vivax* (e.g., Restrepo *et al*. 2011; Nyachieo *et al*. 2005; Orjuela-Sanchez *et al*. 2009).

When faced with the inability to perform whole genome sequencing STRs are time- and cost-effective and easy to develop in the lab and still useful for parentage analysis, relatedness between primary infection and recurrence-relapse isolates, population genetics, and phylogenetic analysis.

6. Another potential strength of STRs might be better coverage of 'non-core' parts of the genome that are often ignored because of the challenge in assaying them. Is this the case? Do the STR markers bring some new genome regions into view?

We thank the reviewer for bringing this to our attention. The *Plasmodium* core genome is stable, while the non-core genome region (e.g., telomeres, centromeres, and sub-telomeres) exhibit high sequence variability, and poor sequencing coverage in the telomeric and centromeric regions make them hard to assemble and map. We accessed the SNP and STR genotyping accuracy using the Pf3K three genetic crosses datasets (https://www.malariagen.net/parasite/p-falciparum-genetic-crosses) and found the non-core genome region displayed higher Mendelian error rates and discordance rates between replicates at both SNP and STR data (S1 Fig; S2 Fig). Therefore, in our study, we only included the core genome in *Plasmodium* but note that this provides thousands of viable, high quality STR markers.

7. There is little attempt to use the AT content differences of P. falciparum vs P. vivax to assess the particular unique strengths of STRs. Are they particularly powerful when applied in AT rich genomes? This might provide unique insights into subtelomeric regions and multi gene families?

We thank the reviewer for this comment. *P. falciparum* has an extremely high AT-content genome, hence STR sequences are extraordinarily abundant throughout the genome, occurring every 2–3 kb (Su *et al*. 1996). *P. falciparum* has a high mutation rate primarily associated with repetitive AT-rich sequences, which pose a great challenge to the study of STRs in *P. falciparum*. Some STRs in *P. falciparum* simply expand and contract rapidly due to frequent errors in DNA replication, which might represent non-functional 'junk DNA' or intrinsically disordered (Davies *et al*. 2017). In our study, we were able to identify high-quality STRs throughout the genome of *P. falciparum* based on our model indicates this approach works on AT-rich genomes, which has, as yet not been demonstrated. However, due to the limitations of short-read sequencing, (low sensitivity and accuracy in subtelomeric regions), the set of STRs revealed in this study will still be incomplete. The use of long-read sequencing data should be able to provide additional functional STRs into subtelomeric regions and multigene families in the future. We have added this point to the discussion (Line 564).
