# Supplementary material for: Population-level genome-wide STR discovery and validation for population structure and genetic diversity assessment of Plasmodium species
Source: PLoS Genet. 2022 Jan 10;18(1):e1009604. doi: 10.1371/journal.pgen.1009604 (PMC8782505; doi:10.1371/journal.pgen.1009604)
Supplement: Attachment — Submitted filename: STR manuscript second review.pdf [file pgen.1009604.s035.pdf]

The authors have responded thoughtfully to the reviews and now include a more comprehensive web-based R Shiny application and improve the supporting evidence for the accuracy of their calls given the many challenges of assaying them accurately. The inclusion of Pf3K genetic crosses data gives a stronger framework for interpretation as a new data analysis that supports the authors' filtering strategy.

Particularly, and important improvement is there new information for each STR locus in the Rshiny tool: the gene name, gene link to PlasmoDB, AlphaFold (Jumper *et al*. 2021) predictions link for the coding STRs, and the STR multivariable logistic regression model parameter as searchable by the user.

In terms of biological discovery and novel knowledge gained, this remains more of a tool development/description. It is helpful the authors have improved the baseline description and include much more context for the reader to understand/appreciate the improved utility of STRs.

The title states "higher resolution population structure". There are some examples in the data that support this explicitly, but in general the data are more nuanced and this title is not necessarily a generality of the STRs, but may offer advantages over SNP-based methods in some cases. The authors are still vague about what is gained in which circumstances, and in which cases it would be wise to rely only on STRs vs a companion to SNPs. The response to reviewers states: "further highlight the benefits of STRs" without noting specifically the relative strengths and weaknesses (or potential for hidden cases to the user in which weaknesses or biases could be present). Doing so would strengthen the manuscript.

Especially with respect to the question of whether the marker itself is under selection would have a big impact on the result and interpretation. Their new lines in the discussion considering expression STR will make the point that this is work in progress and could have a significant impact on how STRs might have a unique perspective on evolution that must be considered.
