# Supplementary material for: Population-level genome-wide STR discovery and validation for population structure and genetic diversity assessment of Plasmodium species
Source: PLoS Genet. 2022 Jan 10;18(1):e1009604. doi: 10.1371/journal.pgen.1009604 (PMC8782505; doi:10.1371/journal.pgen.1009604)
Supplement: Attachment — Submitted filename: Response to reviewers.pdf [file pgen.1009604.s036.pdf]

We thank all of the three reviewers for their suggestions and we address all of their queries below, point by point.

Reviewer 1.

The authors have carefully considered and responded to each of my original comments. The inclusion of genetic cross data is a great addition. My original concerns about the validation has been mostly addressed and the updates have added clarity. I particularly appreciate the lengthy addressing of comments regarding the circularity of the approach. I also appreciate the availability of code from the work making the analysis of new data feasible.

(a) The remaining issue I have is with the novelty of the work. The work reanalyzes data from a large-scale effort where microsatellites have also been called. Notably, the prior work focused on SNPs and indels, did not differentiate between other indels and microsats, and did not greatly delve into using indels to inform population structure or selection all areas focused upon here. The use of microsats to infer population structure of malaria parasites is very well established, they are widely used albeit often as much smaller panels, and localized patterns of selection (i.e. reduced expected heterozygosity) have been previously exploited to characterize positive selection. Conversely, there is great value is a robustly and specifically called set of microsatellite markers, and the authors do a wonderful job of demonstrating that they are powerful for understanding population structure and recent selection. The work is well placed in the context of other research, though a clearer presentation as to why we need a novel approach for calling microsats, and where the current data are an improvement over either genome-wide SNPs or smaller panels of microsatellites would address this.

We thank the reviewer for this comment. We have updated our introduction and discussion to further make these points (Line 88; Line 93; Line 532).

We have described the novelty of our work in the introduction and discussion of the manuscript, by highlighting the following aspects:

1) We report the largest to date *in silico* STR typing study in *P. falciparum* and *P. vivax* field samples, which is a much more challenging proposition in terms of data analysis compared to previous studies but also with substantial gains in understanding of STR population genetics in the largest population-based *Plasmodium* cohorts analysed for genome-wide STRs.
2) We developed a novel method for the prediction of the quality of STRs which can be used in other large-scale datasets, for any other species with a reference genome, generating a first ever catalogue of genome-wide *Plasmodium* STRs.
3) We performed the largest analysis to date to compare the performance of genome-wide STRs data and SNPs data for the following aspects in *Plasmodium* analysis: delineation of population structure, genetic diversity, and genetic differentiation metrics.
4) This novel catalogue of genome-wide STRs in *Plasmodium* has been made available in the R Shiny application PlasmoSTR, serving as a valuable resource for future studies.

We have emphasized the reasons for developing a novel approach for calling microsatellites in the discussion (Line 512).

We have described the improvements in this manuscript over either genome-wide SNPs or smaller panels of microsatellites in *Plasmodium* studies in the introduction and discussion (Line 88; Line 99; Line 501; Line 532).

Reviewer 2.

The authors have provided a thorough response to my comments and those of the other two reviewers. The validation of the STR typing on the sexual cross data from the Pf3K project, in particular, instills much greater confidence in the accuracy of this approach.

(a) The manuscript still fails to strongly connect on several fronts in terms of the utility of the findings. For example, Figure 3 still provides no evidence that STRs provide a higher resolution of population structure than SNPs, despite this claim comprising much of the title. Sup Figures 8 and 9 show this, but the use of NJ trees to evaluate populate structure in a sexually recombining eukaryote is not an apt or rigorous way to assess this.

We thank the reviewer for this comment.

Figure 3 was mainly describing how the STR data recapitulates the broad geographical structure of the SNP data and does not highlight that the STRs provide a higher resolution of population structure than SNPs.

We agree that this manuscript is more of a tool development/description and have now updated the title to make this clearer.

In the Supplementary Data Figures 8 and 9, we explored the local levels of parasite population structure and found at some local sites, the STR data formed a well-separated and distinct cluster with the different sites, while the SNP data was unable to separate these sites into distinct clusters indicating that STRs can identify more recent (often local) stratification, missed by SNP data (S10 Fig; S11 Fig; S14 Fig).

STRs are complex molecular sequences that are largely ignored in phylogenetic reconstruction. In this study, phylogenetic trees were constructed by the neighbor-joining method (Saitou and Nei 1987) based on the genetic distance for both STRs and SNPs. We decided to focus on the neighbor-joining method for several reasons: (1) the NJ-based method can handle missing data and is thus useful in phylogenetic studies in which data sets often contain missing loci for some samples; (2) the NJ method is widely used in SNPs and INDEL polymorphisms in *Plasmodium* and can represent accurate population structure (Tagliamonte *et al.* 2020; Mathema *et al.* 2020; Ahouidi *et al.* 2021; Osborne *et al.* 2021). In this application, the NJ tree can be regarded as a type of population clustering diagram as opposed to a precise representation of the evolutionary history of the parasite populations. We also exchanged the usual genetic distance measure used for SNPs in NJ methods with Bruvo's distance (Bruvo *et al.* 2004) for the STRs. Bruvo's method considers mutation processes, utilizing a stepwise mutation model appropriate for STRs resulting in a more appropriate genetic distance estimate.

(b) Given the new capacity to perform sexual crosses in humanized mice, it could be interesting to relate this STR genotyping capacity to the capacity to fine-map QTL signals. So, in the discussion of the Pf3K cross progeny validation, it would be useful to note how many STRs were successfully genotyped and were segregating in each cross relative to SNP markers. Reporting the average MER % does not give a sense as to how much value STRs could add to this application.

We thank the reviewer for this comment, and we have now calculated the number of segregating sites between pairwise samples for both STRs and SNPs and added this to the manuscript (Line 198, Line 694, and S4 Fig).

(c) As reviewer 1 notes, the Redmond et al. paper undertook a genome-wide analysis of STRs for the purpose of understanding clonal transmission dynamics. This HIPSTR-based approach could be very exciting for assessing the age of clonal lineages, or bifurcations in their transmission history, due to the expected faster rate at which de novo STR mutations should accumulate relative to SNPs. As a reviewer, I don't think it's fair to think to heap significant new analyses on a manuscript after it's already been revised once (and therefore I do not expect the authors to undertake an analysis of the Redmond data), but this is an example of an application for which this approach could uncontroversially outperform SNPs. Perhaps the authors could allude to this potential application.

We thank the reviewer for this comment. We have added this point to the discussion (Line 538).

<u>**Reviewer 3.**</u>
The authors have responded thoughtfully to the reviews and now include a more comprehensive web-based R Shiny application and improve the supporting evidence for the accuracy of their calls given the many challenges of assaying them accurately. The inclusion of Pf3K genetic crosses data gives a stronger framework for interpretation as a new data analysis that supports the authors' filtering strategy.
Particularly, and important improvement is there new information for each STR locus in the Rshiny tool: the gene name, gene link to PlasmoDB, AlphaFold (Jumper et al. 2021) predictions link for the coding STRs, and the STR multivariable logistic regression model parameter as searchable by the user.

(a) In terms of biological discovery and novel knowledge gained, this remains more of a tool development/description. It is helpful the authors have improved the baseline description and include much more context for the reader to understand/appreciate the improved utility of STRs.
The title states "higher resolution population structure". There are some examples in the data that support this explicitly, but in general the data are more nuanced and this title is not necessarily a generality of the STRs, but may offer advantages over SNP-based methods in some cases. The authors are still vague about what is gained in which circumstances, and in which cases it would be wise to rely only on STRs vs a companion to SNPs. The response to reviewers states: "further highlight the benefits of STRs" without noting specifically the

relative strengths and weaknesses (or potential for hidden cases to the user in which weaknesses or biases could be present). Doing so would strengthen the manuscript.
Especially with respect to the question of whether the marker itself is under selection would have a big impact on the result and interpretation. Their new lines in the discussion considering expression STR will make the point that this is work in progress and could have a significant impact on how STRs might have a unique perspective on evolution that must be considered.

We thank the reviewer for this comment. We agree that this manuscript is more of a tool development/description and have now updated the title to make it more appropriate.

We have now added the relative strengths and weaknesses of STRs to the discussion (Line 532; Line 543) and further state the importance of expression STR analysis (Line 580).
