# Supplementary material for: Population-level genome-wide STR discovery and validation for population structure and genetic diversity assessment of Plasmodium species
Source: PLoS Genet. 2022 Jan 10;18(1):e1009604. doi: 10.1371/journal.pgen.1009604 (PMC8782505; doi:10.1371/journal.pgen.1009604)
Supplement: Attachment — Submitted filename: STR manuscript review.docx [file pgen.1009604.s033.docx]

The authors present interesting and well-done work to use short tandem repeat (STR) markers for population genetic analysis, the first large-scale application in malaria parasites. Their approach is thorough: using cutting edge bioinformatics tools, developing a set of multi variate regression models to predict STR quality, wet-lab crosschecks of their predictions a detailed direct comparison to the standard SNP-based methodologies for a range of population parameters. Their write-up is comprehensive and interesting, providing strong context useful for a general audience and citations. It is impressive that the authors have generated a web-based R Shiny application, PlasmoSTR and make extensive supporting information available.

STRs have many appealing features for genetic analysis but they have not been readily accessible to users due to the many challenges of assaying them accurately, lack of quality controls and a general unknowns with respect to their performance. The authors have gone to great effort to build a tool that is easily accessible and user-friendly. They then applied it to a massive dataset (3K *P. falciparum*, 174 *P. vivax*) whole genome sequences. It is likely that this baseline information will be heavily used/cited not only by malaria researchers but by those who would emulate this same optimization template for their own species.

In terms of biological discovery and novel knowledge gained, this work as presented is less forceful, not as coherent and generally lacks a strong take-home message. The authors can do more to make these points and argue more effectively that STRs in malaria parasites can be a game-changer, particularly by being more detailed about what the particular strengths and weaknesses of this approach are; there are a wide range of variables and parameters that are only cursorily considered but are very important.

Detailed comments:

The title states “higher resolution population structure”. There are some examples in the data that support this explicitly, but in general the data are more nuanced. For some kinds of questions STRs may offer an advantage over SNP-based methods. It would strengthen the value of this work to specify more clearly what is gained in which circumstances. It seems more likely that STRs can be a useful companion to SNPs.

There are a wide range of variables involved that are both fascinating and possibly confounding. The authors do a good job of mentioning them but fall short of making definitive statements in most cases. It would be helpful to the reader to better understand the information content breakdown for specific types of evolutionary questions. Mono vs Poly tracts (and type of poly), length of the STR, position in the genome, position in the gene (if it impacts a gene).

Especially with respect to the question of whether the marker itself is under selection would have a big impact on the result and interpretation. A preponderance of the STRs resides in promotors. The authors note that this large sequencing dataset is not accompanied by RNA-seq data. Do they have other ways (other datasets) to ascertain their impact? Indeed, the functional implications of STRs are very interesting on their own, but how that interfaces with being high quality genome-wide markers needs more consideration.

For genome wide genetic differentiation measures, what does generally “higher values” than SNPs accomplish? Does that indicate better information?

The authors make interesting observations about the value of STRs in local/recent evolution (or maybe better describes as monitoring of genome variants, clonal expansions, recrudescence vs reinfection. These are VERY important for malaria control monitoring and could be more strongly focused on, and in consideration if that might make these markers less useful for deeper evolution studies.

Another potential strength of STRs might be better coverage of ‘non-core’ parts of the genome that are often ignored because of the challenge in assaying them. Is this the case? Do the STR markers bring some new genome regions into view?

There is little attempt to use the AT content differences of P. falciparum vs P. vivax to assess the particular unique strengths of STRs. Are they particularly powerful when applied in AT rich genomes? This might provide unique insights into subtelomeric regions and multi gene families?
